# Structural and Catalytic Properties of S1 Nuclease from *Aspergillus oryzae* Responsible for Substrate Recognition, Cleavage, Non–Specificity, and Inhibition

**DOI:** 10.1371/journal.pone.0168832

**Published:** 2016-12-30

**Authors:** Tomáš Kovaľ, Lars H. Østergaard, Jan Lehmbeck, Allan Nørgaard, Petra Lipovová, Jarmila Dušková, Tereza Skálová, Mária Trundová, Petr Kolenko, Karla Fejfarová, Jan Stránský, Leona Švecová, Jindřich Hašek, Jan Dohnálek

**Affiliations:** 1 Laboratory of Structure and Function of Biomolecules, Institute of Biotechnology CAS, v. v. i., Biocev, Vestec, Czech Republic; 2 Department of Agile Protein Screening, Novozymes A/S, Bagsvaerd, Denmark; 3 Department of Fungal Strain Technology, Novozymes A/S, Bagsvaerd, Denmark; 4 Department of Protein Biochemistry and Stability, Novozymes A/S, Bagsvaerd, Denmark; 5 Department of Biochemistry and Microbiology, University of Chemistry and Technology, Prague, Czech Republic; Griffith University, AUSTRALIA

## Abstract

The single–strand–specific S1 nuclease from *Aspergillus oryzae* is an archetypal enzyme of the S1–P1 family of nucleases with a widespread use for biochemical analyses of nucleic acids. We present the first X–ray structure of this nuclease along with a thorough analysis of the reaction and inhibition mechanisms and of its properties responsible for identification and binding of ligands. Seven structures of S1 nuclease, six of which are complexes with products and inhibitors, and characterization of catalytic properties of a wild type and mutants reveal unknown attributes of the S1–P1 family. The active site can bind phosphate, nucleosides, and nucleotides in several distinguished ways. The nucleoside binding site accepts bases in two binding modes–shallow and deep. It can also undergo remodeling and so adapt to different ligands. The amino acid residue Asp65 is critical for activity while Asn154 secures interaction with the sugar moiety, and Lys68 is involved in interactions with the phosphate and sugar moieties of ligands. An additional nucleobase binding site was identified on the surface, which explains the absence of the Tyr site known from P1 nuclease. For the first time ternary complexes with ligands enable modeling of ssDNA binding in the active site cleft. Interpretation of the results in the context of the whole S1–P1 nuclease family significantly broadens our knowledge regarding ligand interaction modes and the strategies of adjustment of the enzyme surface and binding sites to achieve particular specificity.

## Introduction

Nucleases of the S1–P1 family [1, 2] can be found in fungi, plants, protozoan parasites and, interestingly, in some bacteria. They are zinc dependent nucleases/3’nucleotidases active on both RNA and DNA with acidic or close to neutral pH optima. They act as phosphoesterases cleaving the P–O3’ bond and producing 5‘mononucleotides as end products. A cluster of three zinc ions coordinates the substrate/product scissile phosphate and the reaction mechanism utilizes water activated by Zn^2+^ ions as a nucleophile [2]. Their native role usually lies in nucleotides/nucleosides scavenging [3], specific apoptotic functions [4, 5] and in symbiont (pathogen)–host interactions [6]. This enzyme class is generally substrate sequence–nonspecific and representatives from different types of organisms realize substrate binding via a variation of the same basic approach. Even if several studies already addressed ligand binding in this nuclease family exhaustive experimental evidence regarding ligand binding principles in this family is still missing.

S1 nuclease from *Aspergillus oryzae* (EC 3.1.30.1; NCBI sequence ID: XP_001818636; S1–P1 nuclease family in Pfam, PF02265) is an extracellular, single–strand–specific, sugar non–specific, Zn^2+^–dependent, fungal nuclease with 3'–mononucleotidase activity [7] with a pH optimum in range 4.0–4.3 [8]. Mature S1 nuclease is a glycoprotein with two N–glycosylation sites. It is composed of 267 amino acids with a molecular mass of 29.1 kDa (about 35 kDa when fully glycosylated). It can be utilized in many biochemical methods; in one of its applications it is used as an analytical tool for determination of the secondary structure of nucleic acids [9]. The most likely natural role of S1 lies in scavenging of phosphate and nucleotides [2].

Structures of three S1 nuclease homologs are known: P1 from *Penicillium citrinum* [10, 11], TBN1 from *Solanum lycopersicum* [12] and AtBFN2 from *Arabidopsis thaliana* [13, 14]. The fungal single–strand–specific nuclease P1 shares the highest sequence identity with S1 (51%). Despite close sequence and fold similarity, there are several important differences between these two nucleases. P1 has slightly more basic pH optimum (around 5.5). It prefers 3'AMP over RNA and single–stranded DNA (ssDNA) and can cleave also double–stranded DNA (dsDNA) although at a significantly reduced level [2]. S1 is more active on single–stranded nucleic acids with preference for ssDNA over RNA and 3’AMP [2]. Both nucleases prefer 3'–ribomononucleotides over 3'–deoxyribomononucleotides, but with slightly different base preference [2, 7]. Tomato bifunctional nuclease TBN1 (27% sequence identity with S1) and bifunctional nuclease AtBFN2 (31% sequence identity with S1) belong to the plant nuclease I family, in which they form a sub–family called plant S1–like nucleases [5]. Interestingly, despite the same fold and active site composition there are important differences in the catalytic activity of S1, TBN1, and AtBFN2. Along with differences in pH optima and base and sugar preferences the main difference lies in the fact that TBN1 is active against multiple types of nucleic acids (NAs) including structured DNA and highly stable viroid RNA [15]. AtBFN2 is also capable of cleaving dsDNA although it prefers single–stranded NAs. S1 and P1 on the other hand highly prefer single–stranded NAs. This raises questions regarding the key factors causing such major distinction between plant and fungal nucleases. Finally, the role of amino acids adjacent to the active site in the hydrolysis of NAs has not been fully explained either.

Nuclease and nucleotidase products are competitive inhibitors of the members of the S1–P1 nuclease family, however, no potent selective inhibitors for this family are known. In this study, we focus on the structure–function relationship in S1 nuclease, as a model system of the family, in complexes with varied ligands, in order to investigate substrate recognition, draw conclusions regarding its catalytic properties and provide structure–based background for design of inhibitors with potential applications in biotechnology and medicine.

Along with the wild type of S1 nuclease (S1wt), biochemical properties of four structure–based mutants (S1D65N, S1K68N, S1N154A, and S1N154S) were studied in this work. Analyses of the S1 reaction mechanism and substrate/inhibitor interactions are based on six structures of S1wt and one structure of S1D65N in complex with ligands obtained at pH 4.2, 5.5, and 6.5.

## Results

Seven structures of S1 nuclease in complexes with various ligands and at different pH were obtained. The following paragraphs describe the structural features of S1 nuclease common for all the currently reported structures and the most important details in each of them.

### General features of S1 nuclease structure

#### Crystal packing, protein fold, and post–translational modifications

Crystals of S1 nuclease were obtained under four distinct crystallization conditions with the main difference being in their pH (4.2, 5.5, and 6.5). Depending on the particular condition and the type of ligand in co–crystallization, S1 crystallized with five different unit cells and in four different space groups (Table 1). In six of the seven structures reported here at least one N–linked N–acetyl–D–glucosamine moiety (GlcNAc; the first saccharide unit left after deglycosylation using Endo F1) participates in crystal contacts.

**Table 1:** Structures of S1 nuclease–crystallization conditions, ligands, selected data collection and structure refinement statistics.

Structure title	5FB9 –unoccupied	5FBA–phosphate	5FBB–inhibitors	5FBC–remodeled	5FBD–nucleotidase products	5FBF–nuclease products	5FBG–mutant with products
**PDB ID; figure**	5FB9; Fig 3A	5FBA	5FBB; Fig 3B and 3D	5FBC; Fig 3E	5FBD; Fig 3C and 3F	5FBF; Fig 3H and 3I	5FBG; Fig 3G
**Crystallization condition**	Optimized Index No. 70, pH 5.5	Index No. 70, pH 5.5	Index No. 54, pH 6.5	Index No. 70, pH 5.5	Optimized Index No. 40, pH 4.2	Optimized Index No. 40, pH 4.2	Index No. 70, pH 5.5
**Co–crystallization partner**	dGua	None	5'AMP	dA(pS)dA	dC(pS)dC	5'dCMP	d(GC)_6_
**Important ligands**	None (water)	Pi	2x Pi, 2x 5'AMP, 2x Na	5'dAMP(S)	Pi, dCyt	2 x 5’dCMP	2x Pi, 2x dCyt, dGua
**Space group**	*P*1	*P*2_1_	*P*1	*P*2_1_	*P*2_1_2_1_2_1_	*P*2_1_2_1_2_1_	*P*3_1_21
**Unit–cell *a*, *b c*, (Å)**	43.2, 48.6, 65.5	41.8, 62.3, 48.0	42.8, 47.6, 62.6	41.9, 62.6, 48.2	43.0, 62.4, 84.1	53.7, 62.4, 62.8	106.8, 106.8, 127.9
**Unit–cell *α*, *β*, *γ* (°)**	107.4, 90.1, 105.7°	90, 106.7, 90	106.4, 90.1, 106.3	90, 107.0, 90	90, 90, 90	90, 90, 90	90, 90, 90
**Resolution range (Å)**	43.05–1.50 (1.53–1.50)	45.97–1.80 (1.84–1.80)	35.51–1.75 (1.78–1.75)	46.14–1.75 (1.78–1.75)	30.08–1.75 (1.78–1.75)	44.25–1.04 (1.06–1.04)	37.47–1.97 (2.02–1.97)
***R***_**meas**_	0.084 (0.264)	0.090 (0.504)	0.137 (0.791)	0.120 (0.785)	0.072 (0.504)	0.097 (0.394)	0.144 (0.750)
**Mean *I/σ(I)***	9.2 (3.8)	11.7 (2.2)	8.1 (2.6)	10.7 (2.0)	13.6 (2.1)	10.7 (2.7)	9.3 (2.0)
**Completeness (%)**	88.2 (54.6)	95.3 (69.0)	95.8 (94.7)	99.8 (99.8)	95.2 (72.2)	94.5 (54.5)	99.6 (97.6)
***R***_**work**_	0.149	0.152	0.145	0.131	0.152	0.111	0.158
***R***_**free**_	0.177	0.199	0.188	0.181	0.212	0.135	0.187
**R.m.s.d. bonds (Å)**	0.015	0.016	0.017	0.017	0.017	0.013	0.016
**R.m.s.d. angles (°)**	1.647	1.688	1.728	1.678	1.706	1.695	1.598
**Ramachandran plot (%)**^a^							
**Favored**	98.37	96.99	97.01	98.17	97.74	97.93	97.77
**Outliers**	0	0	0	0	0	0	0

Values in parentheses are for the highest resolution shell. dGua stands for 2'–deoxyguanosine, dCyt for 2'–deoxycytidine and Pi for phosphate ion. Details regarding crystallization are summarized in Table A in S1 File. Data processing and refinement statistics are summarized in Table B in S1 File.

^a^As calculated by MolProbity [17].

**Fig 1:**
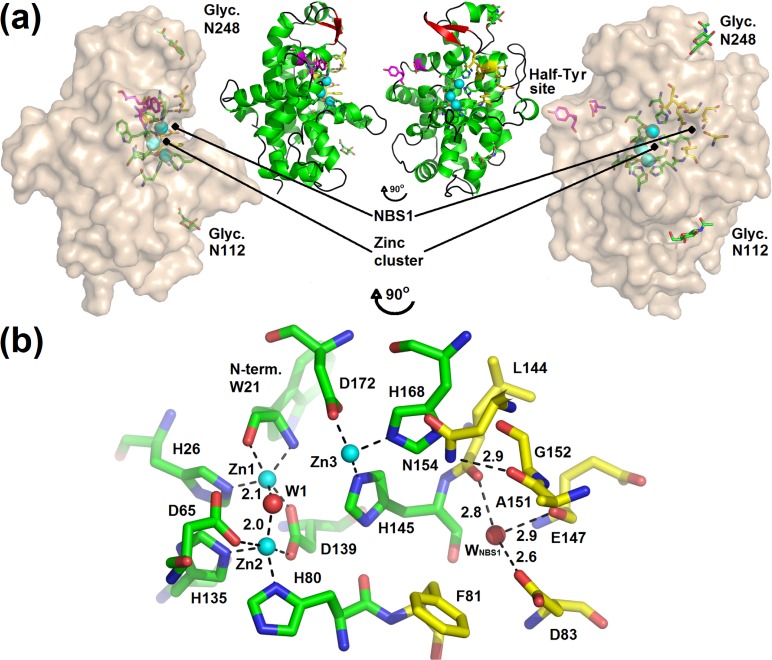
Overview of the structure of S1 nuclease. **(a)** S1 nuclease represented by its surface with important sites in sticks. The secondary structure representation is shown in the inset (helices are colored green, β–strands red, loops black). The catalytic zinc ions are shown as light blue spheres. Glycans are shown as sticks (carbon–green) and labelled according to the modified residues. Residues involved in the zinc cluster coordination are shown as sticks (carbon–green), residues forming NBS1, (also known as Phe–site) as sticks with carbon in yellow. Residues forming the Half–Tyr site are shown as sticks (carbon–magenta). NBS1 and the Half–Tyr site delimit the extent of the active site cleft. The graphics was created based on the structure 5FBF–nuclease products with removed 5’dCMP. **(b)** The active site of S1 nuclease. The color scheme is the same as in (a). Residues involved in the zinc cluster coordination and formation of NBS1 are labelled. The activated water molecule is labelled as W1, the water molecule present inside NBS1 as W_NBS1_. Important interaction distances of W1, W_NBS1_ and the distance of Asn154N^δ2^ to Gly152O are given in Å. The graphics was created based on the structure 5FBA–phosphate, alternative without PO_4_. Phe81 is shown in alternative A (occupancy factor 0.7). Molecular graphics were created using *PyMOL* (Schrödinger, LLC).

**Fig 2:**
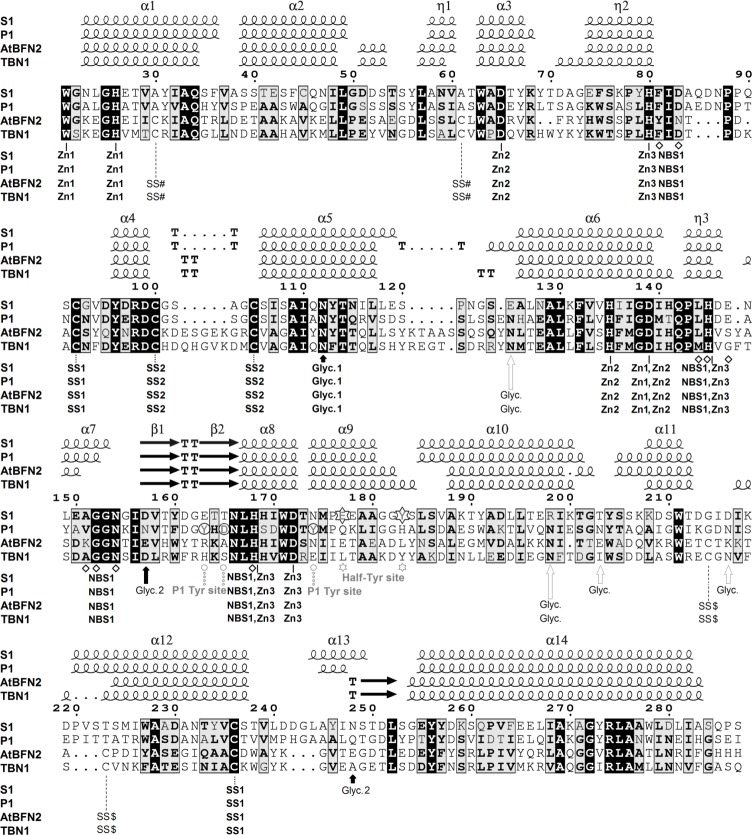
Comparison of amino–acid sequences and secondary structures of S1, P1, TBN1, and AtBFN2 nucleases. Secondary–structure labeling for S1 nuclease is shown: α–helices as α, 3_10_–helices as η, β–sheets as β, β–turns as T. Residues coordinating Zn^2+^ ions are marked by solid line, cysteine residues forming disulfide bridges by dotted line, S1 nuclease glycosylation sites conserved in P1 nuclease (Glyc. 2 is conserved in position in space, not in sequence) are marked by black arrows. Glycosylation sites not present in S1 are marked by empty arrows. Residues forming the Nucleoside binding site 1 (NBS1) are marked by diamonds. Labels of features conserved in all four nucleases are in bold letters. The Half–Tyr site of S1 nuclease is marked by stars. The Tyr site of P1 nuclease is marked by circles. Secondary structure was assigned by ENDscript [16] based on the structures of S1 5FBF–nuclease products, P1 nuclease–PDB ID: 1AK0 [11], AtBFN2 –PDB ID: 3W52 [13] and TBN1 –PDB ID: 3SNG [12]. The figure was created using ESPript [16] and manually edited.

S1 nuclease has the phospholipase C/P1 nuclease–like fold stabilized by two disulfide bridges. The mature protein chain starts with Trp21 (the first twenty residues form a signal sequence and are cleaved off in the process of maturation) and ends with Ser287. Two N–glycosylation sites were identified at Asn112 and Asn248. All samples used for crystallization were deglycosylated using Endo F1 (Figure A in S1 File) and so only one GlcNAc can be present at each glycosylation site in the structures reported here. The main features of the structure of mature S1 nuclease are shown in Fig 1A and important structural attributes are also marked in the amino acid sequence alignment in Fig 2.

#### Active site

The main features of the active site follow all the other members of this family with known structure. The active site is located in a surface cleft (Fig 1) and composed of the obligatory catalytic trinuclear zinc cluster supplemented by Lys68 and the nucleoside binding site 1 (NBS1; also called Phe site in the previous studies [11, 12]). The zinc ions are coordinated by the N–terminus main chain (Trp21) and side chains of several histidine and aspartic acid residues. The cluster connects distant parts of the protein chain (Figs 1 and 2). Organization of the zinc cluster was described in detail in our previous study of TBN1 [12]. NBS1 is an open pocket on the enzyme surface in close proximity to the zinc cluster. NBS1 in S1 nuclease is composed of the Phe81 side chain and the Ala151–Gly152 peptide bond, both providing stacking interactions to a nucleobase, and of Asp83 providing hydrogen bonding. These four amino acids are exposed to the solvent and create the NBS1 opening. The bottom of the pocket is composed of the main chain carbonyls of Leu144 and Glu147 and residue His145. In vacant NBS1 and in some complexes there is a water molecule (W_NBS1_) linking together the two main chain oxygen atoms and the side chain oxygen of Asp83. NBS1 is completed by the side chain of Asn154, which stabilizes the pocket through a hydrogen bond to the main chain oxygen of Ala151. N^δ2^ of Asn154 can act as a donor in hydrogen bonding to the sugar moiety of a ligand. His168 (involved in the coordination of Zn3) can also be considered a part of NBS1. Residues involved in formation of the active site are shown in Fig 1B and marked in Fig 2.

### Structures of complexes

A structure of S1 nuclease with unoccupied active site, two binary complexes, three ternary complexes, and one quaternary complex are further described. To provide evidence for binding of the discussed ligands composite omit maps were calculated for each important ligand and are included in the supplementary data (Figure B to Figure H in S1 File).

#### Structure with unoccupied zinc cluster

**Fig 3:**
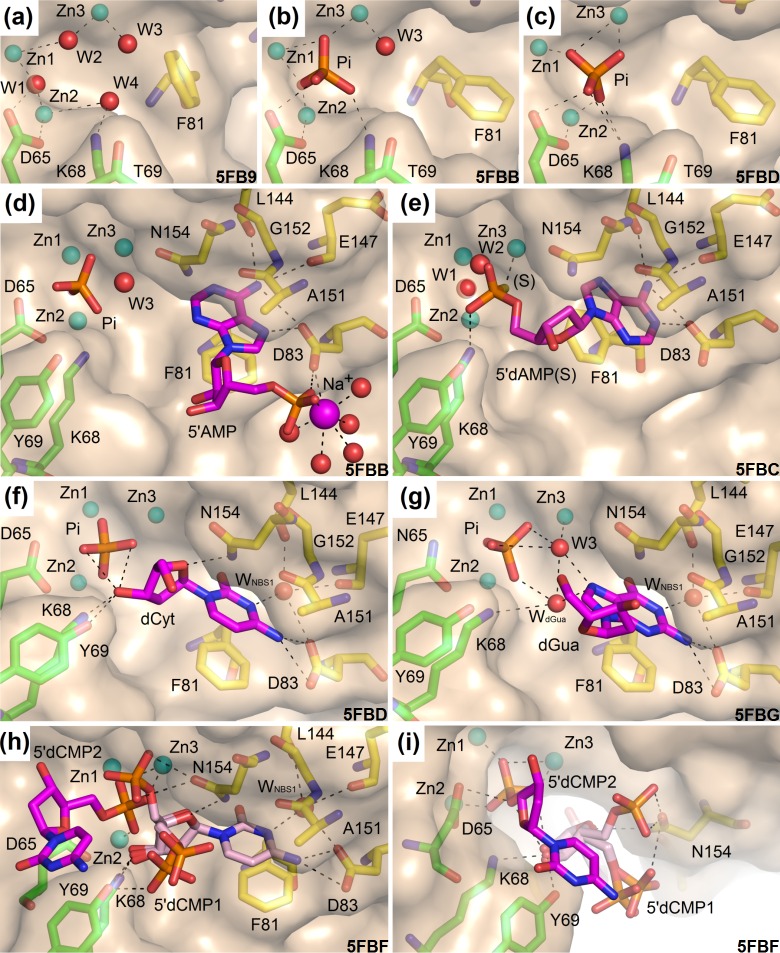
Observed binding of the ligands in S1 nuclease structures. The catalytic zinc ions are shown as light blue spheres. Asp65 (Asn65 in the case of the mutant) is shown in sticks (carbon–green). Other residues involved in the zinc cluster coordination are not shown. Lys68 and Tyr69 interact with ligands and are shown as sticks (carbon–green). Residues forming NBS1 are shown as sticks (carbon–yellow). Important water molecules are shown as red spheres and phosphate ions as orange/red sticks. Selected interactions are shown as black dashed lines. Molecular graphics were created using *PyMOL* (Schrödinger, LLC). PDB ID of each structure is shown. **(a)** Binding of water molecules in the unoccupied zinc cluster in the structure 5FB9 –unoccupied (pH 5.5). **(b)** The first binding mode of the phosphate ion in the structure 5FBB–inhibitors (pH 6.5). 5’AMP is excluded from the graphics for clarity. **(c)** The second binding mode of phosphate ion in the structure 5FBD–nucleotidase products (pH 4.2). All water molecules are displaced and none of the oxygen atoms of the phosphate ion occupies the original positions of water molecules. dCyt is excluded from the graphics for clarity. **(d)** The inverted deep binding mode of 5’AMP in the complex 5FBB–inhibitors (pH 6.5). Water W_NBS1_ is replaced by the inhibitor. The zinc cluster is occupied by a phosphate ion. **(e)** The binding mode of 2'–deoxyadenosine 5'–thio–monophosphate in the complex 5FBC–remodeled (pH 5.5). The thiophosphate moiety binds outside the zinc cluster interacting only with Zn3 using its sulfur atom (marked as S), with Lys68 by the oxygen atom and through a water network with Asn154. In this deep binding mode W_NBS1_ is replaced. Notice the same position of the adenine amino group, similar to the position described in (d), but an entirely inverted orientation of the base. NBS1 is remodeled to the extended form. **(f)** The observed binding mode of 2'–deoxycytidine (carbon–magenta) in the complex 5FBD–nucleotidase products. The cytosine moiety interacts with the protein using several types of interactions. Its π–conjugated system interacts with Phe81 and the peptide bond between Ala151 and Gly152. It has a direct polar interaction with the side chain of Asp83 and several water–mediated interactions, including involvement of water inside the NBS1 site. The 2’–deoxyribose moiety binds close to the zinc cluster and interacts directly with Lys68N^ζ^, Tyr69O^η^, Asn154N^δ2^, and the phosphate ion. A similar binding occurs in the complex 5FBF–nuclease products (for the dCyt moiety of 5’dCMP) and in the complex 5FBG–mutant with products. **(g)** The observed binding mode of 2’–deoxyguanosine (carbon–magenta) in the complex 5FBG–mutant with products, chain B. Binding of the pyrimidine–like part of guanine mimics the orientation of cytosine inside NBS1 as shown in panel (f). N7 of the imidazole–like part is involved in the water network (W3 and W_dGua_) connecting this atom to Zn3, the phosphate ion, and Lys68N^ζ^. Asp154N^δ2^ can interact with the π–conjugated electrons of the guanine moiety. **(h)** The observed binding mode of two molecules of 5’dCMP in the complex 5FBF–nuclease products (pH 4.2). The binding mode of the first 5’dCMP (carbon–pale pink) is almost identical with binding of 2’–deoxycytidine in the case of the complex 5FBD–nucleotidase products (panel f). The phosphate moiety is disordered and interacts either with Asn154 or with Lys68 and Tyr69. **(i)** The phosphate moiety of the second 5’dCMP in the complex 5FBF–nuclease products (carbon–magenta) binds in the zinc cluster in the second binding mode (as phosphate ion in 5FBD–nucleotidase products, panel f). The cytosine moiety likely interacts with Tyr69O^η^ (hydroxyl group) through its π–conjugated system.

The crystal of the structure 5FB9 –unoccupied was obtained at pH 5.5. The structure contains two protein chains in the asymmetric unit. Coordination of the zinc ions is completed by four water molecules W1–W4 (Fig 3A and Figure B in S1 File). This structure represents a possible resting state of the enzyme.

#### Complex with phosphate ion

The crystal of the structure 5FBA–phosphate was obtained at pH 5.5. A phosphate ion inside the zinc cluster in the first binding mode is modeled with occupancy 0.5 (Figure C in S1 File). Phosphate ion is a product of 3’–mononucleotidase activity and simultaneously an inhibitor of S1 nuclease. This structure brings evidence for one of its binding modes and for its capability to bind inside the active site. It binds in the same mode as in the structure 5FBB–inhibitors (the next paragraph).

#### Complex with two independent inhibitors: phosphate ion and 5’AMP

This complex brings clear evidence for the fact that two inhibitors can bind to the active site at the same time and without direct competition. Each of the two protein chains in the asymmetric unit binds one phosphate and one molecule of adenosine 5'–monophosphate (5’AMP) in the same binding mode. The crystal of the structure 5FBB–inhibitors was obtained at pH 6.5. Phosphate is one product of the 3’–mononucleotidase activity, 5’AMP is a product of nuclease activity. Both ligands are also inhibitors. Phosphate is present inside the zinc cluster, 5’AMP occupies the NBS1 site in an inverted orientation with respect to the standard substrate/product binding (the ribose moiety is far from the zinc cluster, Fig 3D).

One of the phosphate oxygen atoms replaces the water molecule W1 and binds almost symmetrically between Zn1 and Zn2 (Fig 3B). Another oxygen atom binds asymmetrically between Zn1 and Zn3 replacing W2. W3 stays in the same position as in the unoccupied zinc cluster and W4 is displaced (compare Fig 3A and 3B for water replacement/displacement). The phosphate ion also interacts with Asp65 and Lys68 (Fig 3B and Figure D in S1 File). We further refer to this binding mode as the first binding mode of the phosphate ion, similar to the previous observations in TBN1 (PDB ID: 4JDG [18]) and AtBFN2 (PDB ID: 4CXV [14]).

The phosphate moiety of 5’AMP interacts not with the zinc cluster but instead with Asp83O^δ2^ of NBS1 through a sodium ion (Fig 3D). The placement of the phosphate moiety is slightly affected by a crystal contact–a direct interaction with symmetry–related Glu42O^δ1^ (Figure D in S1 File). There is no direct interaction of the ribose moiety with the protein. The adenine base binds inside NBS1 in a position almost exactly above and parallel to the side chain of Phe81. The water molecule W_NBS1_ is substituted by the amino group of the adenine base, which enables a deeper penetration of the ligand, and therefore we denote this binding mode as deep. Hydrogen bonding to Asp83 is realized through the Hoogsteen face of adenine (Fig 3D).

#### Complex with inhibitor 5’dAMP(S) in remodeled NBS1

The crystal of the structure 5FBC–remodeled was obtained at pH 5.5. The structure contains one molecule of 2'–deoxyadenosine–5'–thiomonophosphate (5’dAMP(S)) in the active site (Fig 3E). Here, one molecule of the inhibitor binds simultaneously to both NBS1 and the zinc cluster. This complex also shows an alternative binding of the adenosine moiety (when compared with the binding mode present in 5FBB–inhibitors) in which NBS1 undergoes remodeling.

The thiophosphate moiety of 5’dAMP(S) interacts with the zinc cluster from the side of NBS1, binding only to Zn3 by its thiol group and to Lys68N^ζ^ by an oxygen atom. The deoxyribose moiety does not interact directly with protein and is disordered. The adenine moiety inside NBS1 is in the deep binding mode but different from that in the structure 5FBB–inhibitors (compare Fig 3D and 3E). The amino group still substitutes W_NBS1_ as in 5FBB–inhibitors but the adenine moiety interacts with Asp83 using the Watson–Crick face (Fig 3E and Figure E in S1 File).

**Fig 4:**
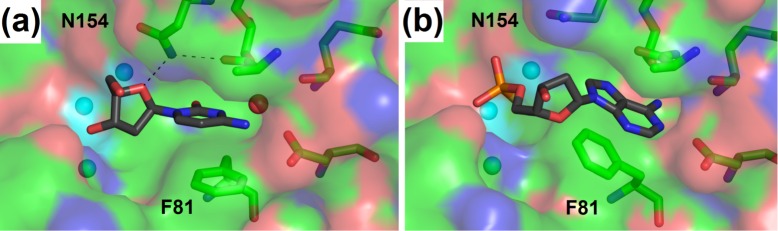
Remodeling of NBS1. The Nucleoside binding site 1 of S1 nuclease is represented as sticks (carbon–green). The solvent accessible surface of protein, color–coded by atom types, is shown. Zinc ions are shown as light blue spheres. Ligands are shown as sticks (carbon–grey). Selected interactions are shown as black dashed lines. The protein orientation in both panels is identical; changes of Phe81 and Asn154 can be seen. **(a)** The compact form of NBS1 with shallow base binding observed in the structure 5FBD–nucleotidase products. **(b)** The extended form of NBS1 with deep base binding observed in the structure 5FBC–remodeled. Molecular graphics were created using *PyMOL* (Schrödinger, LLC).

Here NBS1 is remodeled when compared to all the previously published structures of the S1–P1 nuclease family members. The phenyl ring of Phe81 is rotated closer to Zn3 and makes an almost parallel stacking interaction with the adenine moiety of the ligand. The H–bond between the main chain of Ala151 and the side chain of Asn154 is broken, and the side chain of Asn154 is displaced in order to avoid clashes with the base and the 2’–deoxyribose moiety of the ligand. Remodeling of NBS1 will be described further and is also shown in Fig 4.

#### Complex with products of 3’–nucleotidase activity: phosphate and 2’–deoxycytidine (dCyt)

The structure contains one phosphate ion and one molecule of dCyt in the active site (Fig 3C and 3F). This ternary complex (further called 5FBD–nucleotidase products) with the phosphate ion and dCyt represents binding of products of 3’nucleotidase activity and was obtained at pH 4.2. Both, the phosphate ion and the deoxyribose moiety of dCyt, are found in novel binding modes, observed here for the first time.

Two of the phosphate oxygen atoms bind directly to Zn1 and Zn2 (Fig 3C) and the ion also interacts with Asp65 and Lys68. All water molecules of the previously unoccupied zinc cluster (Fig 3A) are displaced but none of them is substituted by any oxygen atom of the phosphate ion (Fig 3C). We further refer to this interaction as the second binding mode of the phosphate ion.

The cytosine moiety of dCyt binds in NBS1 via stacking interactions with the side chain of Phe81 and the peptide bond Ala151–Gly152 (Fig 3F). The pyrimidine ring of dCyt is not placed exactly above the Phe81 side chain but slightly shifted out of NBS1. The deep position in NBS1 remains occupied by W_NBS1_. Therefore, this nucleoside binding mode is denoted as shallow. The difference between the deep and shallow binding modes of nucleobase can be seen in Fig 3D and 3F or in Fig 4A and 4B. Asp83 along with W_NBS1_ provide hydrogen bonding interactions to the base. The position of O1 of the cytosine moiety inside NBS1 allows interaction with the π–system of His145 (His145 is not shown in Fig 3F). The deoxyribose moiety binds via O4’ to Asn154N^δ2^. Its O3’ interacts with Lys68N^ζ^ and Tyr69O^η^ and is placed approximately between the two atoms (Fig 3F and Figure F in S1 File).

#### Complex with products of dinucleotide cleavage: two molecules of 5’dCMP

The complex 5FBF–nuclease products was crystallized at pH 4.2 and contains two molecules of 2'–deoxycytidine 5'–monophosphate (5’dCMP) bound in the –1 and +1 positions with respect to the cleaved P–O3’ bond (Fig 3H and 3I, and Figure G in S1 File). This ternary complex represents one of the possible binding states of single–stranded NA in the active site after cleavage.

One molecule of 5’dCMP binds to the active site in a similar way as 2'–deoxycytidine in the case of the complex 5FBD–nucleotidase products described above (compare Fig 3F and 3H). Its phosphate moiety is disordered and present in two main positions (Fig 3H and Figure G in S1 File). The phosphate moiety of the second molecule of 5’dCMP is bound inside the zinc cluster in the second phosphate binding mode (Fig 3H and 3I). Its deoxyribose moiety has no direct contact with the protein but is indirectly connected via a network of water molecules. Its oxygen atom O3’ is positioned in the active site cleft and oriented towards the Half–Tyr site (described further) at a distance of about 12 Å from the side chain of Tyr183. The cytosine moiety is placed close to Tyr69 and it is likely that the hydroxyl group Tyr69O^η^ and the π–conjugated system of Tyr69 interact with the cytosine π–conjugated electrons; its position is slightly affected by a crystal contact (Figure G in S1 File).

#### Quaternary complex of mutant D65N with products of nucleotidase activity in the active site and a nucleoside in the secondary binding site

The complex 5FBG–mutant with products crystallized at pH 5.5 with two protein chains in the asymmetric unit shows the active site after mutation of the key catalytic residue Asp65 to Asn. Products of 3’monunucleotidase activity are bound in the active site: phosphate with 2’–deoxycytidine or 2'–deoxyguanosine. The third important feature is the identification of the secondary binding site in the active site groove based on binding of dCyt.

Asp65 is a part of the active site (Fig 1B) of wt S1 nuclease. The mutant D65N has the same tertiary structure as the wild type. The active site organization is also unchanged, with the only difference being the change of Asp65 to Asn.

**Fig 5:**
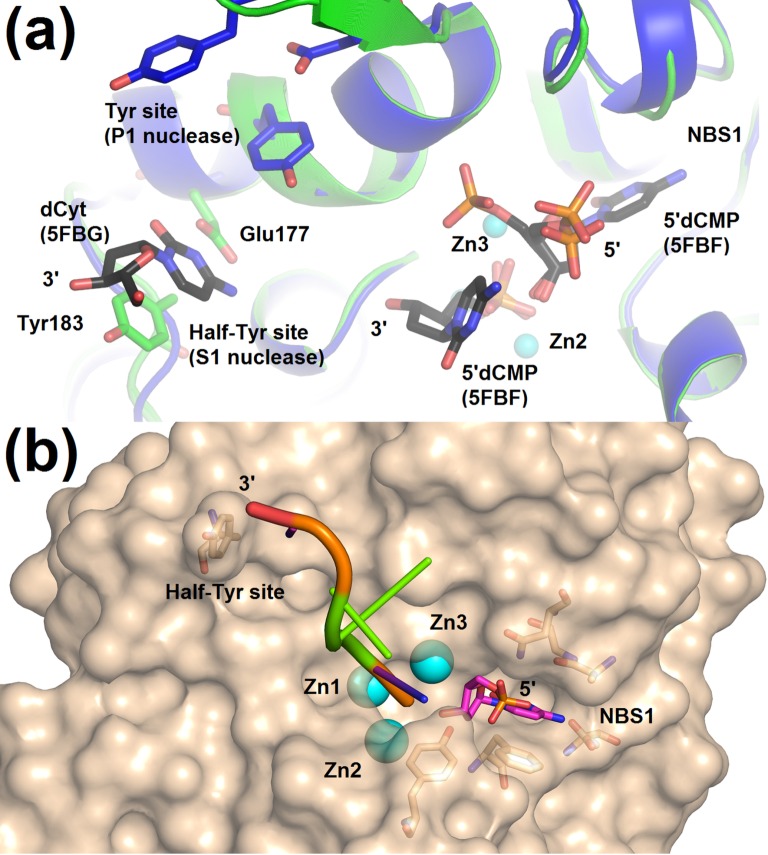
The Half–Tyr site of S1 nuclease and the proposed binding of ssDNA. **(a)** 2'–deoxycytidine (shown as sticks, carbon–magenta) bound in the Half–Tyr site and comparison of the position of the Half–Tyr site of S1 nuclease (carbon–green) with the position of the Tyr site of P1 nuclease (carbon–dark blue) and with respect to the active site. Both secondary sites are positioned at the same end of the active site cleft, albeit on the opposite “banks”. Superposition of the complexes 5FBG–mutant with products, chain A, 5FBF–nuclease products, and of P1 nuclease (PDB ID: 1AK0 [11]) was calculated using the SSM Superpose tool in *Coot* [19]. Residues forming the Half–Tyr site are shown as sticks (carbon–green) and labelled. Residues of P1 nuclease involved in the formation of the Tyr site are shown as sticks (carbon–dark blue) and not labelled. The catalytic zinc ions (light blue spheres) with the phosphate ion (red/orange sticks) inside the zinc cluster are shown only for S1 nuclease. NBS1 is not shown but its position is marked. 2'–deoxycytidine and 5’dCMP binding in the active site cleft are shown (carbon–grey). **(b)** The proposed binding mode of ssDNA in the active site cleft based on the observed interactions of nucleotides and nucleosides in the S1 nuclease structures. The placement of the five nucleotides is based on x–ray structure coordinates except for the two nucleotides shown in green, which were positioned manually with optimized chain geometry in order to demonstrate the possible direction of ssDNA binding. Molecular graphics were created using *PyMOL* (Schrödinger, LLC).

A phosphate ion is bound inside the zinc cluster in the first binding mode in protein chain A (Figure H in S1 File). One molecule of dCyt binds in the active site as in the complex 5FBD–nucleotidase products (Figure H in S1 File). The second molecule of dCyt binds in the secondary binding site (Fig 5A and Figure H in S1 File) composed of Tyr183 providing a stacking interaction for the nucleobase and Glu177 providing hydrogen bonding. We refer to this site as “Half–Tyr site” with respect to the P1 nuclease Tyr site which is composed of two tyrosine residues [11]. The Half–Tyr site is a part of the active site groove (Fig 1A), about 13 Å distant from the catalytic zinc cluster, and exposed to solvent (Figs 1A and 5).

Chain B also contains a phosphate ion in the first binding mode. Simultaneously, one molecule of 2'–deoxyguanosine (dGua) is bound in the active site, interacting with NBS1 and the zinc cluster (Fig 3G and Figure H in S1 File). Interestingly, the placement of the pyrimidine part of the pyrimidine–imidazole ring system of dGua is the same as that of the cytosine moiety in the previous cases (compare Fig 3F and 3G). Additionally, the imidazole part interacts with a water network involving W3 and W_dGua_, which is only present in this structure. These two water molecules mediate its interaction with Zn3, Lys68N^ζ^, Asn154O^δ1^, and the phosphate ion within the zinc cluster. Asn154N^δ2^ likely interacts with the π–system of the guanine moiety. In this binding mode deoxyribose is oriented out of the active site without any direct interaction with the protein.

This complex was crystallized in the presence of self–complementary DNA d(GC)_6_, however, the structure contains single nucleosides. The sample of S1D65N used in crystallization had a small residual activity. Self–complementary d(GC)_6_ easily adopts Z–DNA conformation and can form double helical structures with single–stranded overhangs due to possible shifts in the hybridization process. Such DNA form is then susceptible to cleavage by S1 nuclease.

### Catalytic activity

S1wt under the given reaction conditions (see Materials and Methods) digests RNA with a *V*_lim_ of 28.0 ± 3.0 ΔA_260_/min/μg and a *K*_m_ of 0.16 ± 0.04 mg/ml. It digests ssDNA with a *V*_lim_ of 45.0 ± 3.0 ΔA_260_/min/μg and a *K*_m_ of 0.14 ± 0.03 mg/ml. Activity towards dsDNA under standard reaction conditions is about ten times lower than towards ssDNA.

#### Inhibition by phosphate

Phosphate ion is often present in the active site of the structures reported here. The inhibition effect of inorganic phosphate was tested on ssDNase activity of S1wt under standard reaction conditions. Phosphate inhibits activity by 70% at 10 mM concentration and by 91% at 100 mM concentration.

#### Catalytic activity of the structure–based mutants S1D65N, S1K68N, S1N154A, and S1N154S

**Fig 6:**
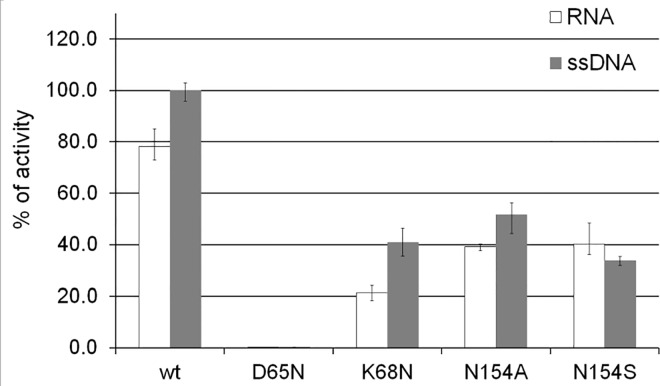
Comparison of the catalytic activity of S1 wild type and mutants. The catalytic activity of S1wt, S1D65N, S1K68N, S1N154A, and S1N154S is shown as a percentage of the S1wt activity on ssDNA.

Residues Asp65, Lys68, and Asn154 are located in the active site and interact with ligands (Figs 1 and 3). Therefore they were identified as targets for structure–based mutational studies. Catalytic properties of the variants of S1 nuclease are reported here, in Fig 6 and Table 2; raw data can be found in Figure I in S1 File.

**Table 2:** Comparison of kinetic parameters of nuclease S1 wild type and its mutants N154S and N154A using ssDNA and RNA as a substrate. Standard deviations are given.

	ssDNA				RNA		
Nuclease S1	*V*_lim_ [ΔA_260nm_/min/μg]	*K*_m_ [μg/μL]	*V*_lim_/*K*_m_ [ΔA_260nm_μL/min/μg^2^]	*K*_S_ [μg/μL]	*V*_lim_ [ΔA_260nm_/min/μg]	*K*_m_ [μg/μL]	*V*_lim_/*K*_m_ [ΔA_260nm_μL/min/μg^2^]
**Wild type**	45 ± 3	0.14 ± 0.03	321	–	28 ± 3	0.16 ± 0.04	175
**N154A**	55 ± 7	0.32 ± 0.06	172	0.3 ± 0.1	17 ± 2	0.14 ± 0.03	121
**N154S**	16 ± 1	0.12 ± 0.02	133	–	23 ± 1	0.39 ± 0.04	59

Under the standard reaction conditions, mutant S1D65N digests both single–stranded substrates roughly at a five hundred–fold lower rate than S1wt (0.16% of S1wt cleavage rate for RNA and 0.19% for ssDNA). Mutant S1K68N shows 27% of the S1wt cleavage rate for RNA and 41% for ssDNA. Mutant S1N154A has 60% of the S1wt cleavage rate for RNA and 52% for ssDNA, and a different mutant of the same residue, S1N154S shows 62% of the S1wt cleavage rate for RNA and 34% for ssDNA. The changes of activity are presented in Fig 6; for clarity all values in Fig 6 are related to the level of the ssDNAse activity being equal 100%.

Kinetics measured for both mutants S1N154A and S1N154S confirm a significant decrease of the catalytic activity expressed as *V*_lim_/*K*_m_, with the residue mutated to serine the effect being more pronounced. The mutant S1N154A shows inhibition by ssDNA (*K*_s_ = 0.3 ± 0.1 mg/ml), as opposed to no inhibition in the mutant S1N154S or in the wild type. Changes of the kinetic parameters are summarized in Table 2.

## Discussion

### Asp65 is critical for the reaction mechanism

The zinc cluster plays a major role in the catalytic mechanism [1, 2, 20]. Residues involved in coordination of the zinc ions are highly conserved in the S1–P1 nuclease family (Fig 2). Asp65 is involved in the coordination of Zn2 and in the reaction mechanism itself [11, 12]. It can play the role of a general base [21] or can assist in proper orientation of the activated water for the nucleophilic attack [11].

For the first time we provide a structure of a mutant at this position. In order not to severely influence the active site organization, Asp was replaced by Asn. The structure of the protein and organization of the active site are unaffected by this mutation and the enzyme can still bind ligands (see structure 5FBG–mutant with products, Fig 3G and Figure H in S1 File). This mutation basically abolishes the enzymatic activity (see Results and Fig 6), from which it follows that Asp65 is critical for the reaction mechanism. This observation supports the mechanism proposed previously [11, 12, 21], in which the water molecule is activated between Zn1 and Zn2 using Asp65 as a general base. The activated water molecule/hydroxide ion (labeled W1 in Fig 3A) acts as a nucleophile in the in–line attack of the phosphorous atom with a consequential inversion of configuration of the scissile phosphate after cleavage.

### Lys68 has multiple roles but is not critical for the reaction mechanism

The role of the residue at position 68 (Lys68 in S1 nuclease, Lys or Arg in the S1–P1 nuclease family, Fig 3) in the reaction mechanism was proposed previously [11, 12, 22] and investigated using quantum chemical calculations [21]. Lys68 interacts with ligands in six of the seven structures of S1 nuclease reported here (Fig 3), therefore we decided to investigate its importance by mutating to Asn. This mutation causes a decrease of activity to about one third of the original level of S1wt (Fig 6).

Based on the observed interactions of Lys68 with the phosphate ion in the active site (Fig 3B, 3C and 3E) and with products (Fig 3B, 3C, 3F, 3H and 3I) and adhering to the previous studies [11, 21] we propose that Lys68 has multiple functions in the active site; it can stabilize binding of a substrate or of an intermediate state of the scissile phosphate and it can also assist in the recycling of the active site (discussed further). Thus the decrease but not an entire cessation of activity of the K68N mutant can be explained given the fact that its role in the mechanism is not central, albeit important in assisting the right positioning and orientation of the molecular moieties during the enzymatic cycle. Moreover, it is not excluded that the side chain of Asn68 in the variant can still fulfill some of the original roles. We propose that altering the nature of the residue 68 can fine–tune the level of activity of these 3’nucleotidases/nucleases.

### Asn154 is important for proper substrate binding but not essential for catalysis

Asn154, as a part of NBS1, is highly conserved in the whole S1–P1 nuclease family (Figure J in S1 File). Its role in the reaction mechanism was proposed previously [12] but the hypothesis was never tested.

Binding of a nucleobase inside NBS1 can be accompanied by a hydrogen bond between Asn154N^δ2^ and O4’ of 2’–deoxyribose. This interaction was observed in the structures 5FBD–nucleotidase products, 5FBF–nuclease products and 5FBG–mutant with products (Fig 3F and 3H), and previously in P1 nuclease (PDB ID: 1AK0 [11]) and AtBFN2 (PDB ID: 4CXO [14]). Based on our structures we designed a mutation, first, to a short apolar side chain (Ala) to disable any possibility of hydrogen bonding and, second, to a shorter polar side chain (Ser). In a simple comparison of the level of activity under identical conditions both mutations (S1N154A and S1N154S) decrease the rate of cleavage to about 60% for RNA and 30–50% for ssDNA as a substrate (Fig 6). This confirms the significance of the Asn side chain for the catalytic mechanism in this position. To discriminate the detailed effect of these mutations, the full kinetics were also measured (Table 2). Despite all the experimental effort our structures of complexes with Asn154 exposed to contacts with the sugar moiety contain only 2’deoxynucleotides, and therefore direct structural interpretation of the kinetic data can be attempted only for ssDNA as a substrate. The decrease of the overall efficiency on ssDNA of the N154A mutant is caused by a higher *K*_m_, which complies with the assumed role of Asn154 in the proper binding of the substrate and is in agreement with the observed interaction Asn154Nδ2 –O4’ in several structures. Removal of the Asn154 side chain leads to a decrease of the affinity of ssDNA substrate in the productive position. The mutation N154S restores the affinity but the enzyme is not capable of the original turnover, which could be caused by an improper orientation of the substrate. The mutant N154A also shows inhibition by substrate in the case of ssDNA, presumably caused by the lack of the Asn154N^δ2^ –O4’ interaction, possibly also leading to non–productive substrate binding.

Changes of *K*_m_ in these two mutants with RNA as substrate follow an inverse pattern compared to ssDNA (Table 2). The lack of structural information for possible ribose–Asn154 interactions impairs any direct interpretation of the data. Clearly, Asn154 plays a role also in RNA substrate binding and in the catalytic mechanism. There are differences compared to the ssDNase activity but the underlying details remain to be determined.

Asn154 is conserved throughout the S1–P1 nuclease family and has an important role in the catalytic mechanism, however it is not essential for activity. In the case of ssDNA it is involved in binding of 2’–deoxyribose and contributes to the formation of a productive enzyme–substrate complex.

### S1 nuclease non–specificity is primarily caused by NBS1 promiscuity

NBS1 (Fig 1B) is responsible for the nucleoside binding at position –1 with respect to the cleaved P–O3’ bond. This site is always present in the S1–P1 nuclease family. NBS1 is capable of accommodating not only unmodified natural nucleobases (this study) but also unrelated compounds (*e*.*g*. an arginine side chain in the case of TBN1 [12]). Promiscuity of NBS1 can be attributed to its capability to undergo remodeling and to offer varied H–bonding patterns.

#### Remodeling of NBS1

The nucleoside binding site 1 in the structures of S1 nuclease occurs in two distinct conformations. We further refer to these conformations as compact NBS1 and extended NBS1 (Fig 4).

Compact NBS1 is present in every structure of the members of the S1–P1 nuclease family published up to date [11–14, 18]. In this form NBS1 maintains the Ala151 main chain–Asn154 side chain H–bond (Fig 3D, 3F, 3G and 3H). The phenyl ring of Phe81 is located near Asp83 and in an almost parallel orientation with the plane of the peptide bond Ala151 –Gly152 (Fig 4A). The compact form of NBS1 enables the formation of a direct H–bond of Asn154N^δ2^ to deoxyribose O4’ of a ligand (Figs 3F and 4A).

Remodeling of NBS1 was reported for P1 nuclease previously [11] but the respective structure was not published. Remodeled NBS1 is present in several structures reported in this study. The complex 5FBC–remodeled contains extended (remodeled) NBS1 (with occupancy factor 0.8 to correspond with the occupancy of the ligand). Partially remodeled NBS1 is also present in the structures 5FB9 –unoccupied and 5FBA–phosphate. To the best of our knowledge, these are the first published coordinates of an enzyme from the S1–P1 nuclease family with remodeled NBS1.

In the extended (remodeled) form of NBS1 (*e*.*g*. in 5FBC–remodeled) the Phe81 phenyl ring is rotated closer to Zn3 to make an almost parallel stacking interaction with the adenine moiety of a ligand (Figs 3E and 4B). The side chain of Asn154 is displaced from its usual position in order to avoid clashing with the ligand; the direct sugar–Asn154 contact is thus disabled (Fig 4B).

The capability of NBS1 to undergo remodeling between the compact and extended forms allows for binding of substrates/products with varying sugar–base conformations and therefore can significantly contribute to the ligand binding promiscuity (Fig 4). One could speculate that NBS1 remodeling could also play its role in the catalytic mechanism especially with respect to various types of substrates, however, structure–function data in support of such hypothesis are missing. The part of NBS1 formed by the Ala151 –Gly152 peptide bond is also flexible. In the presented structures its position changes by up to 1 Å (not shown; based on a superposition of all the reported structures).

The conformational state of unoccupied NBS1 is not clear. Based on the structure 5FBA–phosphate, without any ligand in NBS1, we conclude that in the unliganded state the Asn154 side chain maintains the H–bond with the main chain of Ala151 while the side chain of Phe81 can be found in either of the two observed conformations (Figure C in S1 File).

#### Variable hydrogen bonding patterns

The second important feature responsible for the ligand binding promiscuity of NBS1 is a high degree of freedom in the formation of H–bonds with ligands as observed in the current structures (see Fig 3D to 3H). One of the key features of NBS1 of S1 nuclease is the presence of water W_NBS1_ (Fig 2B and other). Based on the utilization of this water molecule the nucleobase binding mode can be distinguished as shallow (W_NBS1_ is employed, Fig 3F) or deep (W_NBS1_ is replaced by a part of the nucleobase, Fig 3D). In the structures presented here, cytosine and guanine are found in the shallow binding mode, whereas adenine in the deep mode. These observations do not rule out the possibility of different types of binding for all nucleobases, especially when considering the high flexibility of NBS1 and the fact that at least one observed binding mode of adenine (Fig 3D) and the observed interaction of guanine (Fig 3G) are clearly not consistent with the most likely reaction mechanism (the position of the sugar moiety is too far from the zinc cluster). W_NBS1_ is also present in the structures of P1 nuclease (PDB ID: 1AK0 [11]) and AtBFN2 (PDB ID: 4CXO [14]). Both contain thymidine in the shallow binding mode. W_NBS1_ is also present in the structure of TBN1, PDB ID: 3SNG [12], with an arginine side chain bound inside NBS1. Although W_NBS1_ is located almost in the same position in all the known structures of S1–P1 nucleases, slight changes in its position and coordination may result in the existence of different binding modes for different nucleobases in distant members of the S1–P1 nuclease family.

The side chain of Asp83 (Asn in some cases, see Figure J in S1 File) is also involved in hydrogen bonding of a nucleobase. The protonation state of the Asp side chain depends on pH. The possibility of increased p*K*_a_ of the Asp side chain inside NBS1 was discussed in previous studies [11, 12]. An estimated value of p*K*_a_ of Asp83 using the Karlsberg+ server [23] is about 7. The binding modes of ligands observed in the reported complexes support protonation of the side chain of Asp83 as derived from the possible combinations of H–bond patterns in NBS1.

The experimental data within this study clearly show that both the NBS1 remodeling and the variable H–bonding patterns within NBS1 lead to a whole spectrum of ligand binding states of the enzyme. The extended and compact forms of NBS1 were observed in the currently reported structures in these combinations with the deep and shallow binding modes of a nucleobase: compact/deep (5FBB–inhibitors, Fig 3D), compact/shallow (5FBD–nucleotidase products, Figs 3F and 4A; 5FBF–nuclease products, Fig 3H, and 5FBG–mutant with products, Fig 3G) and extended/deep (5FBC–remodeled, Figs 3E and 4B). The combination extended/shallow was not observed, but is not excluded.

### ssDNA binding as implied by structural data

#### The Half–Tyr site

One of the puzzling enigmas of P1 nuclease is the role of its second nucleobase binding site, called the Tyr site [11]. The confusion was caused by the fact that this site was not conserved in the sequences of many P1 homologues including S1 nuclease (Fig 2 and Figure J in S1 File). Although the P1–like Tyr site is not conserved in the S1 amino acid sequence, its role is most likely substituted by an alternative site–the Half–Tyr site. We propose that the role of the Tyr site in substrate binding in P1 is in S1 performed by the Half–Tyr site based on the structure 5FBG–mutant with products, which contains a molecule of dCyt in the Half–Tyr site (Fig 5A and Figure H in S1 File). From the comparison of the P1 and S1 structures it is clear that both sites are located in a similar position with respect to the active site and the active site cleft (Fig 5A). Tyrosine has no base preference and can adopt a wide range of conformations when stacked to nucleobases [24]. Moreover, the Half–Tyr site is entirely exposed to the solvent (Fig 5A). All these properties make this site highly promiscuous and hence useful for an unspecific nuclease, such as S1.

Many of the S1–P1 nuclease family members from fungi contain either the P1–like Tyr site or the S1–like Half–Tyr site (sequence alignment, Figure J in S1 File). The tyrosine residue is in some cases substituted by phenylalanine or histidine.

The Half–Tyr site is also likely present in many plant S1–P1 nucleases (Figure J in S1 File), although in the cases of distant homologues such predictions must be interpreted with caution. In TBN1 Tyr183 is conserved in the sequence (while Glu177 is substituted by Leu) but it is not present in the same position in the enzyme structure and, instead, interacts with one of the glycosylation oligosaccharides. In TBN1 in the position corresponding to the Half–Tyr site of S1 there are positive residues (Lys, Arg) presumably involved in binding of ds substrates [12]. AtBFN2 employs completely different binding sites for single–stranded nucleic acids [14]. These differences underline the great variability of the S1–P1 nuclease family and clearly show that one cannot easily predict the binding sites in these versatile nucleases merely by sequence analysis.

#### Simultaneous binding of –1 and +1 nucleotides of ssDNA in the active site

The ternary complex with two molecules of 5'dCMP as products (5FBF–nuclease products; Fig 3H and 3I) brings the first experimental evidence for the capability of an S1–P1 nuclease to simultaneously bind the +1 and –1 nucleotides (with respect to the scissile phosphate) with clear localization in the enzyme active site (Fig 3H). In the previous studies the general orientation of the cleaved single–stranded substrate was assumed [11, 12, 14]. Here, for the first time the way of the nucleotide chain placement in the active site cleft can be inferred from the structural data. It implies a tight turn of the ssDNA chain bound in the active site with the phosphate moieties of nucleotides -1, +1 and +2 placed at an angle of about 90 degrees. Such geometry is governed by the interactions of the -1 nucleobase with NBS1 and of the phosphate moiety with the zinc cluster.

#### Proposed binding of ssDNA

To the best of our knowledge, the ternary complex of two 5’dCMP molecules with S1 nuclease (5FBF–nuclease products, Fig 3H) brings the first structural evidence about the directionality of ssDNA binding in the active site cleft. Together with the assumed role of the Half–Tyr site (Fig 5A) we can propose the expected binding of ssDNA in the active site cleft of S1 nuclease (Fig 5B). The predicted interaction is characterized by the 90° turn (measured by phosphate ions) in the catalytic center and most likely no contacts between nucleotide +2 and protein. The nucleotide in position +3 partially interacts with the active site cleft and in position +4 binds in the Half–Tyr site.

### Deoxyribose moiety after cleavage can bind at least in two distinct positions

Localization of the O3’ oxygen of the scissile phosphate during the catalytic cycle is a key element in understanding substrate binding, catalysis, and product removal.

**Fig 7:**
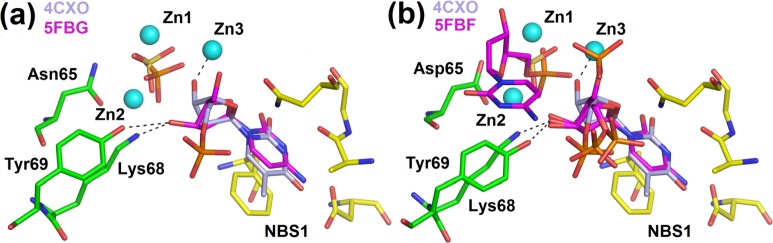
Two types of interactions of the O3’ oxygen with the active site in the S1–P1 nuclease family. Zinc ions are shown as light blue spheres. Asp65 (Asn65 in the case of the mutant) is shown in sticks (carbon–green). Other residues involved in the zinc cluster coordination are not shown. Lys68 and Tyr69 interact with ligands and are shown as sticks (carbon–green). Residues forming NBS1 are shown as sticks (carbon–yellow). Phosphate ion is shown as orange/red sticks, sulfate ion as yellow/red sticks, and selected interactions as black dashed lines. Molecular graphics were created using *PyMOL* (Schrödinger, LLC). **(a)** Comparison of the deoxyribose binding and position of its O3’ in the structure 5FBG–mutant with products with its position in the structure of AtBFN2 (PDB ID: 4CXO [14]). The zinc cluster, residues involved in the interactions and NBS1 are shown only for S1 nuclease. Ligands present in the structure of S1 nuclease are phosphate and dCyt (carbon–magenta). Ligands present in AtBFN2 are sulfate and thymidine 5’–monophosphate (carbon–silver). Notice the difference in the positions of the O3’ oxygens and of the phosphate ion in S1 –excluding the interaction of O3’ with Zn3. **(b)** Comparison of deoxyribose binding and the position of its O3’ in the structures 5FBF–nuclease products and AtBFN2 (PDB ID: 4CXO [14]). The zinc cluster, residues involved in the interactions and NBS1 are shown only for S1 nuclease. Ligands present in the structure of S1 nuclease are two molecules of 5’dCMP (carbon–magenta). Ligands of AtBFN2 are displayed as in panel (a). Notice the difference in the positions of the O3’ oxygens and of the phosphate moiety in the case of S1 which excludes binding of O3’ to Zn3.

In our study, in three cases (5FBD–nucleotidase products, 5FBF–nuclease products, and 5FBG–mutant with products) the O3’ oxygen of deoxyribose of products occurs in the vicinity of the zinc cluster but without direct interaction. The deoxyribose moiety is rotated away from the zinc cluster and the O3’ oxygen binds between Lys68N^ζ^ and Tyr69O^η^. This is in contrast with the situation in the structures of P1 nuclease (PDB ID: 1AK0 [11]) and of AtBFN2 (4CXO [14]), where the O3’ oxygen of the ligands interacts with Zn3 of the cluster. (Fig 7). The structures 5FBD–nucleotidase products (Fig 3F) and 5FBF–nuclease products (Fig 3I), were obtained at pH 4.2, close to the S1 nuclease pH optimum for ssDNAse activity (around pH 4). The structures of P1 nuclease and AtBFN2 with product–like ligands (Fig 7B), were obtained at pH 5.3 and 7.5, respectively. The preference for the second binding mode of phosphate in our two structures may be correlated with pH (compare other structures of the series, Table 1 and Fig 3) and this mode excludes a concurrent interaction of deoxyribose O3’ with Zn3.

Being the only available structures of ternary complexes with both products and at the optimal pH for the respective activity we propose that these can represent the situation in the active site right before the products of the nucleotidase (5FBD) and nuclease (5FBF) activity are released. The phosphate moiety already escaped the tight first binding mode complying with the cleavage step and O3’ of deoxyribose of the nucleotide in position –1 left Zn3 and, instead, interacts with Lys68 and Tyr69.

### Variability of phosphate binding

Inorganic phosphate is a product of 3’nucleotidase activity and, as shown in the Results section, also an inhibitor. Only the first binding mode of phosphate (Fig 3B) was observed in the previous structures of the members of the S1–P1 nuclease family till now [14, 18]. It follows from our results that two main binding modes of the phosphate ion in the active site of the wild type of S1 nuclease can be distinguished (Fig 3B and 3C). In both cases the ion interacts with all three zinc ions, Asp65 and Lys68. The difference lies in the position of its oxygen atoms with respect to the zinc ions (compare Fig 3B and 3C). The first binding mode is realized in this study in the structures crystallized at pH 5.5 and 6.5. The second binding mode is observed in this study only in the case of crystallization at pH 4.2 and is accompanied by binding of 2’–deoxycytidine or 5’dCMP in NBS1 (Fig 3F, 3H and 3I). This implies a certain role of pH in the formation of the interactions of the phosphate ion/moiety with the cluster, however, the influence of the nucleotides/nucleosides present in NBS1 on the phosphate binding mode cannot be neglected and the current results do not allow a simple interpretation.

To the best of our knowledge, an interaction of the phosphate moiety of a mononucleotide/oligonucleotide with the active site in the S1–P1 nuclease family was never observed before. In our structures we observe three different ways of binding of the phosphate moiety of 5’–mononucleotide to S1 nuclease. First, it can mimic the interactions of the free phosphate ion (structure 5FBF–nuclease products, Fig 3I). Second, the thiophosphate moiety of 5’dAMP(S) can interact with the zinc cluster from an outer position (structure 5FBC–remodeled, Fig 3E) and, third, the phosphate moiety can also interact with NBS1 instead of the zinc cluster (structure 5FBB–inhibitors, Fig 3D).

### 5’–mononucleotides can inhibit S1 nuclease via several distinct binding modes

Three types of binding of a 5’–mononucleotide (product and simultaneously inhibitor [2]) in the active site were observed in the structures of S1 nuclease reported here.

In the first case two molecules of 5’dCMP mimic one of the possible arrangements of products after cleavage (structure 5FBF–nuclease products, Fig 3H and 3I, discussed above) and at the same time they show a two–molecule inhibition mechanism. This complex confirms the capability of a 5’mononucleotide to inhibit this nuclease not only as a ligand of NBS1 (–1 position) but also as a ligand of the zinc cluster (+1 position).

In the second case (5FBC–remodeled) the thiophosphate moiety of 5’dAMP(S) interacts with the zinc cluster from an outer position. Contrary to the complex with 5’dCMP, binding of 5’dAMP(S) is marked by the phosphate moiety missing from the zinc cluster with a simultaneous remodeling of NBS1 to the extended form (Figs 3F and 4).

In the third observed case a nucleotide/nucleoside binds in the way that its sugar and phosphate moieties are positioned too far from the zinc cluster to participate in interactions (structures 5FBG–mutant with products, chain B, Fig 3G and 5FBB–inhibitors, Fig 3D). The phosphate moiety either interacts with NBS1 (Fig 3D) or is disordered with no interaction with the protein (Fig 3G). In this type of complexes a phosphate ion can be present inside the zinc cluster at the same time.

We conclude that inhibitors of this nuclease family can utilize the zinc cluster but can also inhibit without direct interactions with the cluster. The most probable binding mode of an inhibitor depends on the nature of the nucleobase, on the presence of phosphate ions and pH. It is likely that a mixture of the different binding modes of a given inhibitor exists in solution and also that new, so far unobserved, binding modes are possible.

### Specificity–related differences between S1 nuclease and other members of the S1–P1 family are defined by the enzyme surface

The fold of S1 nuclease is very similar to those of nucleases P1, TBN1, and AtBFN2 (Fig 2). Residues involved in the coordination of the zinc cluster, the glycosylation site Asn112, as well as two disulfide bridges present in S1 nuclease, are conserved (Fig 2). The main structural differences lie in the facilitation of the secondary substrate binding sites, which largely determines specificity, in the closely related charge distribution on the enzyme surface and in the enzyme shape (Figure K in S1 File). The overall structural features of mature S1 nuclease as well as a comparison with P1, TBN1 and AtBFN2 nucleases are shown in the amino acid sequence alignment in Fig 2.

### Glycosylation has a minor role in S1 nuclease stabilization and does not affect activity

S1 nuclease has two N–glycosylation sites. The Asn112 site is conserved in all members of this family with known structure (Fig 2) and most probably also in the whole family (Figure J in S1 File). Under normal conditions, oligosaccharides account for about 18% of the mature protein mass [2]. Elimination of the major part of oligosaccharides by Endo F1 neither decreases activity (Figure A in S1 File) nor causes the enzyme to unfold (presented 3D structures) or aggregate (verified by DLS, Figure A in S1 File). This is in contrast with the behavior of the so far studied plant homologues TBN1 and AtBFN2, losing stability and solubility upon deglycosylation [11, 25]. A slight decrease of its thermal stability is the only observed effect caused by S1 nuclease deglycosylation (Figure L in S1 File). It should be noted that the only so far studied bacterial member of the S1–P1 nuclease family, M1 nuclease from *Mesorhizobium loti*, naturally lacks glycosylation and yet is stable and active [6].

## Conclusions

1. The three–dimensional structure of S1 nuclease revealed new specific features including the Half–Tyr site and NBS1 remodeling and confirmed conservation of some of the main features of the S1–P1 family, including the helical fold with the central cluster of three zinc ions involving the N–terminal Trp26.
2. Asp65, coordinating one of the zinc ions, is essential for activity. Its mutation to asparagine does not affect the active site organization but abolishes activity. Lys68 on the NBS1–distal side of the active site is not essential but its mutation decreases activity. Asn154 of NBS1 is not essential for activity but, amongst others, is involved in interactions with deoxyribose of ssDNA substrate. Kinetic data for ssDNA activity of the Asn154 mutants can be explained by changes in substrate affinity.
3. NBS1 can accommodate a nucleobase in different binding modes–shallow and deep. In the shallow mode a nucleobase interacts with water W_NBS1_; in the deep mode this water molecule is replaced by a part of the nucleobase. In addition to the shallow and deep binding modes, upon ligand binding NBS1 can undergo substantial remodeling depending on the character of the ligand. The two most extreme observed states of NBS1 –the compact and the extended forms, distinguished by conformations of Phe81 and Asn154 –bind their ligands in positions differing in the orientation of the base plane by about 30 degrees and in the position of the (deoxy)ribose ring. NBS1 can thus bind substrate in two or more different conformations.
4. A new accessory nucleotide binding site, named Half–Tyr site, was identified by ligand binding. It utilizes Tyr183 and Glu177 and is located about 13 Å from the zinc cluster downstream along the active site cleft. Its existence explains the absence of the Tyr site, previously found in P1 nuclease, in sequences of many fungal members of the S1–P1 nuclease family. Most likely all nucleases of this family require a second ligand binding site apart from NBS1, which is located across the zinc cluster. Such site can be realized as a more specific nucleobase binding site (Tyr site, Half–Tyr site) preferred by single–strand–specific enzymes, or by positive surface patches for double strand activity (TBN1, [12]). Based on our structural data a placement of an ssDNA strand in the active site cleft of S1 nuclease is proposed with the implication of a 90° turn of the chain in the zinc cluster.
5. Four different ways of the interaction of phosphate with S1 nuclease were observed, three of them for the first time in the S1–P1 nuclease family. There are two different binding modes of phosphate inside the zinc cluster; the phosphate moiety of a nucleotide can interact with the zinc cluster via binding only to Zn3 and not inside the cluster; it was also observed to interact with NBS1. These phosphate binding modes are likely affected by pH and possibly also by the binding mode of the carrying or co–binding nucleotide or nucleoside in the active site.
6. Five different binding modes of nucleotides and nucleosides in the active site were observed distinguished by the position and orientation of the ligand base and by placement of the phosphate moiety. Two of them represent binding of both products after cleavage of a dinucleotide or of a mononucleotide. The remaining ones are considered inhibitor binding. In four cases two molecules, either of the same ligand or two different ligands, were bound in the active site. The wide range of the utilized interactions shows the true diversity of the nucleic acid binding patterns in the S1–P1 family, which explains its universality and non–specificity. It also broadens the set of interaction points which can be utilized in inhibitor design.

## Materials and Methods

### Expression and purification

#### Cloning of S1 nuclease wild type

Gene for S1 nuclease (*A*. *oryzae* RIB40 gene Ao090001000075) was amplified from *A*.*oryzae* strain IFO4177 by primers 5’–GACGCGGCCGCACCATGCCGCGCTTACTCCC and 5’–GACGCGATCGCTCAAGAGGGCTGACTCG having overhangs with recognition for restriction endonuclease sites, NotI and SgfI, respectively. The amplified DNA (band of 988 base pairs) was digested with restriction endonucleases NotI and SgfI and the resulting 977–base–pair product was cloned into the corresponding restriction sites of an expression vector.

#### Construction of mutated variants of S1 nuclease

Genes of all variants were generated by spliced overlap extension (SOE) polymerase chain reaction (PCR) with flanking primers 5′–AACTGGGGATCCACCATGCCGCGCTTACTCC (forward) and 5’–ACCAGGTCTTAAGTCAAGAGGGCTGACTCGCAATC (reverse) and hybrid primers. The resulting nuclease variant genes were cloned into an expression vector as a BamHI–AflII fragment using standard molecular biology techniques.

#### Expression and purification

After verification by DNA sequencing, constructs were transformed into protoplasts of *Aspergillus oryzae* for expression driven by the TAKA amylase promoter. The transformed strain of *A*. *oryzae* was typically grown for 4 days at 30°C in DAP–4C medium (11 g MgSO_4_–7H_2_O, 1 g KH_2_PO_4_, 2 g citric acid, 20 g dextrose, 10 g maltose, 5.2 g K_3_PO_4_–H_2_O, 0.5 g yeast extract, 0.5 ml trace metals, 0.5 g CaCO_3_, 23 ml of a 50% solution of (NH_4_)_2_HPO_4_, 33 ml of a 20% solution of lactic acid per liter). The fermentation broth was sterile filtered to remove fungal hyphae. Salts and other low molecular weight solutes were removed by ultrafiltration. 1 M Tris–HCl, pH 7.5 was added to the resulting retentate to a final concentration of 25 mM. pH and ionic strength were determined to be within the acceptable range for anion exchange chromatography. The chromatography was then conducted with an ÄKTA Prime instrument (Amersham Biosciences). Briefly, the protein was bound to a column with 20 ml Q Sepharose High–Performance pre–equilibrated with 25 mM Tris–HCl, pH 7.5. After a thorough wash with the equilibration buffer, the bound protein was eluted from the column with a linear NaCl gradient (0–0.5 M) in the equilibration buffer over ten column volumes. Collected fractions containing pure nuclease, as estimated by SDS–PAGE, were pooled. All purification steps were carried out at room temperature.

### Deglycosylation

All samples were enzymatically deglycosylated in order to improve crystallizability. Two different deglycosylation enzymes were tested on S1wt: Endoglycosidase F1 (Endo F1) from *Elizabethkingia miricola* (EC 3.2.1.96) and α–Mannosidase (α–Mann) from *Canavalia ensiformis* (EC 3.2.1.24). All reactions were done in 100 mM sodium acetate, pH 4.5 with 1 mM ZnCl_2_. For 0.1 mg of S1 0.1 μg of Endo F1 or α–Mann was used. Reactions were carried out at 37°C for 1 h. Cleavage was monitored by SDS–PAGE (Figure A in S1 File). Oligomeric state of both samples after deglycosylation was monitored by dynamic light scattering and isoelectric focusing (Figure A in S1 File). The activity of S1 nuclease deglycosylated by Endo F1 (S1–Endo F1) was compared to the activity of the fully glycosylated version (Figure A in S1 File). S1D65N was deglycosylated only with Endo F1 prior to crystallization. All chemicals were purchased from Sigma–Aldrich.

### Nuclease activity

The activity of all S1 nuclease variants was measured towards commercially available isolated nucleic acids. The reaction mixtures contained 50 μl of native DNA from calf thymus (dsDNA), heat–denatured DNA from calf thymus (ssDNA) or RNA from torula yeast (concentration 1 mg/ml in 0.1 M sodium acetate buffer, pH 4.5 containing 50 mM NaCl), and 50 μl of the enzyme diluted in the same buffer. All reactions were carried out at 37°C for 5 min. These assay settings are referred to as standard reaction conditions. Each reaction was stopped by adding 250 μl of 96% ethanol. The mixture was vortexed and incubated at –20°C for 20 min. The precipitated undigested substrate was centrifuged (22 000 x g, 20 min, 4°C) and the absorbance of the supernatant was measured at 260 nm. Each measurement was performed in triplicate. Separate background readings for individual concentration points of all substrates were used in all cases. The inhibitory effect of phosphate was tested by measuring activities towards ssDNA in the presence of inorganic phosphate at two different concentrations (10 mM and 100 mM). The reaction was performed as described above. All chemicals were purchased from Sigma–Aldrich.

#### Calculation of kinetic parameters

Specific activities of S1wt and mutants N154S and N154A were determined using ssDNA or RNA as a substrate. The values of *K*_m_, and *V*_lim_ were calculated using the iterative method for statistical evaluation of deviations in the tool Solver (Microsoft Excel). The following equation was used to calculate the inhibition constant, where [*S*] represents substrate concentration, *v*_0_ initial velocity, *K*_m_ is Michaelis constant, *V*_lim_ maximum velocity and *K*_S_ is substrate inhibition constant [26]:

$$v_{0}=\frac{V_{lim}.[S]}{K_{m}+(1+\frac{\left[S\right]}{K_{S}}).[S]}$$

### Crystallization

For initial crystallization trials fully glycosylated S1wt as well as S1wt deglycosylated with Endo F1 and α–Mann were used. Prior to crystallization, all samples were transferred to 25 mM Bis–Tris pH 6.0 with the addition of 50 mM NaCl by several cycles of concentration/dilution using a Nanosep® centrifugal device with 10 kDa cut–off (Pall Corporation) and concentrated to 25 mg/ml. The Index crystallization screen (Hampton Research) and the hanging drop vapor diffusion method at 18°C with the ratio of the protein to reservoir drop volume 1:1 (0.4 μl + 0.4 μl) were used as the screening setup. Crystals appeared only in the case of S1–Endo F1 and grew in several conditions in 3 to 21 days. The most promising crystals originated from conditions no. 40 (0.1 M Citric acid pH 3.5, 25% w/v Polyethylene glycol 3350), no. 54 (0.05 M CaCl_2_, 0.1 M Bis–Tris pH 6.5, 30% v/v Polyethylene glycol monomethyl ether 550) and no. 70 (0.2 M NaCl, 0.1 M Bis–Tris pH 5.5, 25% w/v Polyethylene glycol 3350). Crystals used in this study were obtained using the original conditions no. 54 and no. 70, the optimized condition no. 40 (0.1 M Citric acid pH 3.8, 25% w/v Polyethylene glycol 3350), and the optimized condition no. 70 (0.05 M CaCl_2_, 0.2 M NaCl, 0.1 M Bis–Tris pH 5.5, 25% w/v Polyethylene glycol 3350) as reservoir solutions. All crystals were obtained using the hanging drop or the sitting drop vapor diffusion methods at 18°C with the ratio of protein to reservoir drop volume 1:1 (0.4 μl + 0.4 μl). For a more precise determination of pH in crystallization drop mixtures of storage buffer with reservoir solutions (without polymer) in ratio 1:1 were prepared. The measured values of pH were within 0.1 unit difference from the values of the corresponding reservoir buffers in the cases of nos. 54 and 70 whereas for the optimized condition no. 40, the resulting value was 4.2 compared to the original 3.8. S1D65N –Endo F1 was crystallized using the condition no. 70 and the same setup as in the case of S1wt.

Crystals of 5FBA–phosphate were obtained without any co–crystallization effort with protein concentration 25 mg/ml. For co–crystallization experiments solutions of ligands were mixed with S1 nuclease in v/v ratio 1:10. The resulting protein concentration was always 22.5 mg/ml. Mixtures were incubated for 1 h at room temperature and then crystallized as described above. Successful co–crystallization was achieved with adenosine 5'–monophosphate (5’AMP) and 2'–deoxycytidine 5'–monophosphate (5’dCMP) as a ligand. The structure 5FB9 –unoccupied was obtained from unsuccessful co–crystallization with 2'–deoxyguanosine (dGua). The final concentration of the above mentioned ligands in the mixtures was 10 mM. Three other complexes were obtained using thiophosphorylated dinucleotides and dsDNA. The final concentration of thiophosphorylated 2'–deoxyadenosine dinucleotide dA(pS)dA in the protein–ligand mixture was 1 mM, of thiophosphorylated 2'–deoxycytidine dinucleotide dC(pS)dC 1.8 mM, and of dsDNA d(GC)_6_ 1.2 mM. The latter three nucleic acids were purchased from Generi Biotech (Czech Republic). Unless stated otherwise, all chemicals were purchased from Sigma–Aldrich. Selected crystallization parameters of the reported structures are given in Table 1. Further crystallization details are summarized in Table A in S1 File.

### X–ray diffraction analyses, structure solution, and refinement

Single crystals obtained from the conditions based on Index no. 40 and no. 70 were equilibrated in reservoir solution containing 20% (v/v) glycerol as a cryoprotectant for 15 seconds. There was no need for the addition of cryoprotectant in the case of crystals from the condition no. 54. All crystals were mounted in round LithoLoops (Molecular Dimensions) of appropriate size and vitrified in liquid nitrogen. X–ray diffraction data for 5FBD–nucleotidase products were collected at 120 K using a Gemini Enhanced Ultra diffractometer with an Enhanced Ultra copper (Cu) source and an Atlas CCD detector (Agilent Technologies). Diffraction data were processed using CrysAlis^Pro^ (Agilent Technologies), and scaled and merged using Aimless [27] from the CCP4 suite [28]. Data for 5FB9 –unoccupied were collected at the beamline P13 of the synchrotron radiation source DESY: PETRA III, Hamburg using a Dectris Pilatus 6M–F detector and a Maatel MD2 micro–diffractometer with mini kappa goniometer at 100 K. All the remaining data were collected at the synchrotron radiation source BESSYII, Helmholtz Zentrum Berlin [29] at 100 K. Data for 5FBA–phosphate, and 5FBF–nuclease products were collected at beamline BL14.1 using a Dectris Pilatus 6M detector and a mini kappa goniometer. Data for 5FBB–inhibitors and 5FBG–mutant with products were collected at the beamline BL14.2 using a MAR Mosaic CCD 225 detector and a MAR Research Desktop Beamline goniometer. Data for 5FBC–remodeled were collected at the beamline BL14.3 using a MARmosaic CCD 225 detector and a MAR Research dtb goniometer. Data collected at DESY: PETRA III and BESSYII were processed either using XDSGui [30] or XDSapp [31], scaled using CORRECT and merged using Aimless [27], or alternatively processed using iMosflm [32] and scaled and merged using Aimless [27]. Selection of the processing programs was based on the quality of data statistics and electron density. Data collection and processing statistics are given in detail in Table B in S1 File. Selected statistics are reported in Table 1.

The phase problem for the first structure (5FBA–phosphate) was solved by molecular replacement in MOLREP [33] using the structure of P1 nuclease as a model (PDB ID: 1AK0 [11]). The phase problem for all the other structures was solved using the protein chain of 5FBA–phosphate as a model. All structures were built using Coot [34] and refined in REFMAC5 [35] using restrained refinement. Restraints for nucleotides and nucleosides were generated using the Grade Web Server (grade.globalphasing.org). Standard restraints of the CCP4 library [28] were used for all the other ligands and moieties. *R*_free_ was used as a cross validation method in the refinement. Atomic displacement parameters (ADPs) were refined as isotropic for all structures except 5FBF–nuclease products, for which ADPs were refined as anisotropic due to the atomic resolution of the structure. Hydrogen atoms in riding positions generated by REFMAC5 [35] were used in all refinements. Automatically generated local NCS was used in the cases of 5FB9 –unoccupied, 5FBB–inhibitors and 5FBG–mutant with products. The quality of each structure was assessed using the set of the validation tools in Coot [34], the validation services provided by wwPDB [36] and MolProbity [17]. Detailed refinement statistics are given in Table B in S1 File. Selected statistics are reported in Table 1. Coordinates and structure factors were deposited in the Protein Data Bank under accession numbers: 5FB9 (5FB9 –unoccupied), 5FBA (5FBA–phosphate), 5FBB (5FBB–inhibitors), 5FBC (5FBC–remodeled), 5FBD (5FBD–nucleotidase products), 5FBF (5FBF–nuclease products), and 5FBG (5FBG–mutant with products).

## Supporting Information

S1 File Supporting information with additional Materials and Methods, Figures, and Tables. (PDF)

S1 PDBvalidationreports Validation reports for the reported PDB entries. (ZIP)
