# Supplementary material for: Structural and Catalytic Properties of S1 Nuclease from Aspergillus oryzae Responsible for Substrate Recognition, Cleavage, Non–Specificity, and Inhibition
Source: PLoS One. 2016 Dec 30;11(12):e0168832. doi: 10.1371/journal.pone.0168832 (PMC5201275; doi:10.1371/journal.pone.0168832)
Supplement: S1 File — (PDF) [file pone.0168832.s001.pdf]

Supporting information

Structural and Catalytic Properties of S1 Nuclease from *Aspergillus oryzae* Responsible for Substrate Recognition, Cleavage, Non–specificity, and Inhibition

Tomáš Kovaľ[1*], Lars H. Østergaard[2], Jan Lehmbeck[3], Allan Nørgaard[4], Petra Lipovová[5], Jarmila Dušková[1], Tereza Skálová[1], Mária Trundová[1], Petr Kolenko[1], Karla Fejfarová[1], Jan Stránský[1], Leona Švecová[1], Jindřich Hašek[1], and Jan Dohnálek[1*]

[1] Laboratory of Structure and Function of Biomolecules, Institute of Biotechnology CAS, v. v. i., Biocev, Vestec, Czech Republic
[2] Department of Agile Protein Screening, Novozymes A/S, Bagsvaerd, Denmark
[3] Department of Fungal Strain Technology, Novozymes A/S, Bagsvaerd, Denmark
[4] Department of Protein Biochemistry and Stability, Novozymes A/S, Bagsvaerd, Denmark
[5] Department of Biochemistry and Microbiology, University of Chemistry and Technology, Prague, Czech Republic

* Corresponding authors

E–mail: koval.tomas@gmail.com (TK), jan.dohnalek@ibt.cas.cz (JD)

Materials and methods

SDS–PAGE

The SDS–PAGE monitoring of S1 nuclease deglycosylation was performed under non–reducing conditions using an XCell SureLock™ mini–cell electrophoresis system, pre–cast polyacrylamide NuPAGE® Bis–Tris mini gels with 4–12% gradient, and the Mark12™ Unstained Standard (Life Technologies Corp.). Electrophoresis was performed according to the manufacturer's instructions. 5 µg of each sample was loaded on the gel.

Determination of experimental pI

The experimental pI of S1 nuclease wild type and of all deglycosylated versions was determined by isoelectric focusing (IEF) using an XCell SureLock™ mini–cell electrophoresis system, pre–cast 5% polyacrylamide Vertical Novex® IEF Mini Gels pH 3 – 10, and IEF Marker 3–10 (Life Technologies Corp.). Electrophoresis was performed according to the manufacturer's instructions.

Behavior of S1 nuclease in solution

Oligomerization state and behavior of S1 nuclease samples in solution were tested by dynamic light scattering (DLS). DLS experiments were performed using a Zetasizer Nano (Malvern Instruments) and a 45 µl quartz cuvette. All measurements were performed at 18 °C with a protein concentration of 1 mg/ml, in the storage buffer (25 mM Bis–Tris pH 6.0 with addition of 50 mM NaCl).

Nuclease activity

The reaction mixtures contained 50 µl of heat–denatured DNA from calf thymus (ssDNA) or RNA from torula yeast (concentration 1 mg/ml in 0.1 M sodium acetate buffer, pH 4.5 containing 50mM NaCl), and 50 µL of the enzyme diluted in the same buffer. After 5 min at 37 °C the reaction was stopped by adding 250 µl of 96% v/v ethanol. These assay settings are referred to as standard reaction conditions. The mixture was vortexed and incubated at −20 °C for 20 min. The precipitated undigested substrate was centrifuged (22000 x g, 20 min, 4 °C) and the absorbance of the supernatant was measured at 260 nm. Each measurement was performed in triplicate. Separate background readings for individual concentration points of all substrates were used in all cases.

Thermal unfolding using differential scanning fluorimetry

Thermal stability of fully glycosylated S1 nuclease and a sample treated with Endoglycosidase F1 (see deglycosylation details in the main article) was analyzed by differential scanning fluorimetry using a Prometheus NT.48 apparatus and Prometheus NT.48 Series nanoDSF Grade Standard Capillaries (NanoTemper Technologies GmbH). Samples were in the storage buffer (25 mM Bis–Tris pH 6.0 with addition of 50 mM NaCl). Concentration of both samples was about 0.5 mg/ml. Thermal unfolding was performed in the range from 20 °C to 95 °C at a scan rate of 2.5 °C per minute.

Surface electrostatic potential distribution

Surface electrostatic potential distribution was calculated for protonation states at pH 4, 6, and 8.5; pH 4 is close to the pH optimum for nuclease activity, pH 6 is close to the pH optimum for 3'–mononucleotidase activity, and pH 8.5 was chosen as a point of minimal catalytic activity of S1 nuclease. The calculations were done using APBS [1]. Parameter files were created by PDB2PQR 1.8 using the AMBER force field [2]. Protonation states were assigned by PropKa [3].

SUPPLEMENTARY TABLES

Table A. Crystallization of S1 nuclease.

Structure title	Composition of reservoir solution	Protein concentr. [mg/ml]	Co–crystallization partner	Co–cryst. partner concentr. [mM]	Setup	Reservoir volume [µl]
**5FB9 – unoccupied**	0.05 M CaCl2, 0.2 M NaCl, 0.1 M Bis–Tris pH 5.5, 25% w/v Polyethylene glycol 3350	22.5	2'–deoxyguanosine	10	Sitting drop vapour diffusion	70
**5FBA – phosphate**	0.2 M NaCl, 0.1 M Bis–Tris pH 5.5, 25% w/v Polyethylene glycol 3350	25	none	-	Hanging drop vapour diffusion	600
**5FBB – inhibitors**	0.05 M CaCl2, 0.1 M Bis–Tris pH 6.5, 30% v/v Polyethylene glycol monomethyl ether 550	22.5	adenosine 5'–monophosphate	10	Hanging drop vapour diffusion	600
**5FBC – remodeled**	0.2 M NaCl, 0.1 M Bis–Tris pH 5.5, 25% w/v Polyethylene glycol 3350	22.5	thiophosphorylated 2'–deoxyadenosine dinucleotide	1	Hanging drop vapour diffusion	600
**5FBD – nucleotidase products**	0.1 M Citric acid pH 3.8 (pH 4.2), 25% w/v Polyethylene glycol 3350	22.5	thiophosphorylated 2'–deoxycytidine dinucleotide	1.8	Hanging drop vapour diffusion	600
**5FBF – nuclease products**	0.1 M Citric acid pH 3.8 (pH 4.2), 25% w/v Polyethylene glycol 3350	22.5	2'–deoxycytidine 5'–monophosphate	10	Sitting drop vapour diffusion	70
**5FBG – mutant with products**	0.2 M NaCl, 0.1 M Bis–Tris pH 5.5, 25% w/v Polyethylene glycol 3350	22.5	dsDNA: $d(GC)_6$	1.2	Hanging drop vapour diffusion	600

All S1 nuclease samples were deglycosylated using Endoglycosidase F1 from *Elizabethkingia miricola* and transferred to 25 mM Bis–Tris pH 6.0, 50 mM NaCl, prior to crystallization. Crystallization temperature was 18 °C and the ratio of protein to reservoir drop volume was 1:1 (0.4 µl + 0.4 µl) in all cases. 5FB9 – unoccupied and 5FBF – nuclease products were obtained using CrystalQuick 96 Well Sitting Drop Plate (Greiner) sealed with ClearSeal Film™ (Hampton Research). The rest of the crystals were obtained using 24–well VDX Plates sealed with 18 mm x 0.22 mm siliconized circle cover slides and vacuum grease (Hampton Research).

Table B. Data collection and structure refinement statistics.

Structure title	**5FB9 – unoccupied**	**5FBA – phosphate**	**5FBB – inhibitors**
PDB ID	5FB9	5FBA	5FBB
**Data collection statistics**			
End station, detector	DESY: PETRA III P13, Pilatus 6M–F	BESSY II BL14.1, Pilatus 6M	BESSY II BL14.2, MAR CCD 225
Wavelength (Å)	1.00000	0.91841	0.91841
Crystal–detector distance (mm)	285.6	421.4	180.0
No. of oscillation images processed	3599	1798	400
Exposure per image (s)	0.05	1.0	2.6
Oscillation width (º)	0.05	0.1	0.5
Space group	$P1$	$P2_1$	$P1$
Unit–cell parameters (*a, b, c*, in Å; *α, β, γ* in °)	43.18, 48.59, 65.47; 107.4, 90.1, 105.7	41.76, 62.31, 48.00; 90.0, 106.7, 90.0	42.84, 47.64, 62.60; 106.4, 90.1, 106.3
Resolution range (Å)	43.05 – 1.50 (1.53–1.50)	45.97 – 1.80 (1.84–1.80)	46.14 –1.75 (1.78–1.75)
No. of observations	127078 (3881)	65030 (1936)	96158 (5195)
No. of unique reflections	68828 (2125)	20922 (889)	43898 (2371)
Multiplicity (I+= I-)	1.8 (1.8)	3.1 (2.2)	2.2 (2.2)
Data completeness (%)	88.2 (54.6)	95.3 (69.0)	95.8 (94.7)
Average mosaicity (°)	0.12	0.29	0.97
Mean $I/\sigma(I)$	9.2 (3.8)	11.7 (2.2)	8.1 (2.6)
R_{merge}	0.060 (0.187)	0.074 (0.386)	0.102 (0.594)
R_{meas}	0.084 (0.264)	0.090 (0.504)	0.137 (0.791)
$R_{p.i.m.}$	0.060 (0.187)	0.049 (0.318)	0.090 (0.519)
CC1/2	0.993 (0.907)	0.997 (0.381)	0.989 (0.454)
Wilson B	5.8	17.6	9.9
**Refinement parameters**			
No. of reflections (working set)	65463	19742	41645
No. of reflections (test set)	3364	1078	2252
R_{work}	0.149	0.152	0.145
R_{free}	0.177	0.199	0.188
R_{all}	0.150	0.153	0.146
Mean ADP (Å^2)	13.32	21.29	16.10
RMSD bond lengths (Å)	0.015	0.016	0.017
RMSD bond angles (°)	1.647	1.688	1.728
Solvent content (%)	43.5	40.6	39.2
No. of monomers in ASU	2	1	2
The last modelled residue (chain A / chain B)	S287 / Q285	S287	S287 / S287
No. of non–hydrogen atoms in ASU	5047	2284	4705
No. of water molecules in ASU	871	214	439
List of non–water ligands in ASU	6x Zn^{2+}, 2x Na^{+}, 2x Bis–Tris	3x Zn^{2+}, 1x Pi, 1x Glycerol	6x Zn^{2+}, 2x Pi, 2x 5'AMP, 3x Na^{+}, 4x Bis–Tris, 5x Ca^{2+}, 1x PEG MME
Modelled glycosylation (chain A / chain B)	N112, N248 / N112, N248	-, N248	N112, N248 / -, -

**5FBC – remodeled**	**5FBD – nucleotidase products**	**5FBF – nuclease products**	**5FBG – mutant with products**
5FBC	5FBD	5FBF	5FBG
BESSY II BL14.3, MAR CCD 225	Gemini Enhanced Ultra, Atlas CCD	BESSY II BL14.1, Pilatus 6M	BESSY II BL14.2, MAR CCD 225
0.89450	1.54056	0.91841	0.91841
180.0	55.2	200.5	230.0
166	283	2000	120
5.0	30.0	0.2	4.4
1.0	0.5	0.1	1
$P2_1$	$P2_12_12_1$	$P2_12_12_1$	$P3_121$
41.89, 62.59, 48.24; 90.0, 107.0 90.0	43.04, 62.43, 84.12; 90.0, 90.0, 90.0	53.74, 62.39, 62.76; 90.0, 90.0, 90.0	106.76, 106.76, 127.91; 90.0, 90.0, 120.0
46.14 –1.75 (1.78–1.75)	30.08 – 1.75 (1.78 – 1.75)	44.25 – 1.04 (1.06–1.04)	37.47–1.97 (2.02–1.97)
84531 (4544)	81964 (1979)	580610 (6260)	347503 (14849)
24085 (1299)	22221 (903)	96234 (2708)	59852 (4088)
3.5 (3.5)	3.7 (2.2)	6.0 (2.3)	5.8 (3.6)
99.8 (99.8)	95.2 (72.2)	94.5 (54.5)	99.6 (97.6)
0.26	1.22	0.11	1.01
10.7 (2.0)	13.6 (2.1)	10.7 (2.7)	9.3 (2.0)
0.101 (0.663)	0.061 (0.376)	0.089 (0.307)	0.131 (0.645)
0.120 (0.785)	0.072 (0.504)	0.097 (0.394)	0.144 (0.750)
0.064 (0.416)	0.036 (0.331)	0.037 (0.242)	0.058 (0.372)
0.995 (0.657)	0.996 (0.841)	0.996 (0.815)	0.993 (0.686)
11.1	5.1	4.8	10.4
22868	21053	91430	56785
1198	1138	4712	3024
0.131	0.152	0.111	0.158
0.181	0.212	0.135	0.187
0.133	0.153	0.113	0.159
15.05	11.74	8.32	21.93
0.017	0.017	0.013	0.016
1.678	1.706	1.695	1.598
41.1	36.9	32.3	66.2
1	1	1	2
S287	S287	S287	S287 / Q285
2376	2464	2606	4859
268	377	485	652
3x Zn^{2+}, 1x Glycerol, 1x 5'dAMP(S)	3x Zn^{2+}, 1x Pi, 1x dCyt	3x Zn^{2+}, 1x Na^{+}, 2x 5'dCMP	6x Zn^{2+}, 2x Pi, 2x dCyt, 1x dGua
N112, N248	N112, -	N112, N248	N112, N248 / N112, N248

Values in parentheses are for the highest resolution shell. ASU stands for asymmetric unit, Pi for phosphate ion, PEG MME for a fragment of polyethylene glycol monomethyl ether, 5'AMP for adenosine 5'–monophosphate, 5'dAMP(S) for 2'–deoxyadenosine 5'–thio–monophosphate, 5'dCMP for 2'–deoxycytidine 5'–monophosphate, dGua for 2'–deoxyguanosine, and dCyt for 2'–deoxycytidine.

SUPPLEMENTARY FIGURES

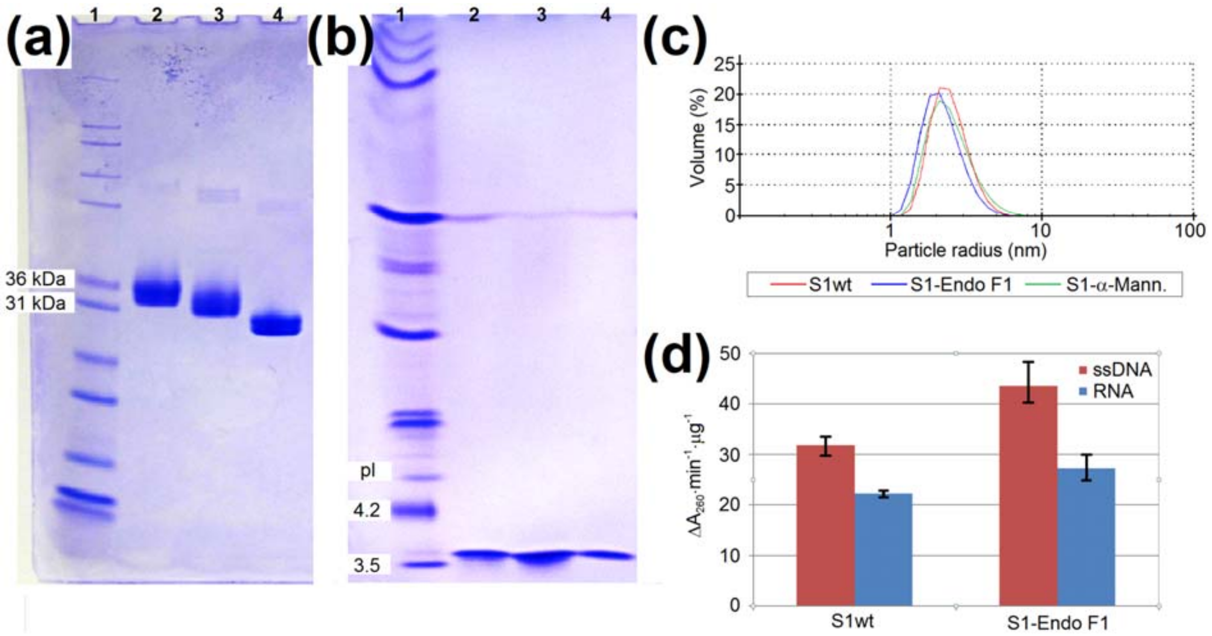

Figure A. Monitoring of S1 nuclease deglycosylation. (**a**) SDS–PAGE analysis. Lane 1: Mark12™ Unstained Standard, lane 2: fully glycosylated S1wt, lane 3: S1wt treated by α–Mannosidase from *Canavalia ensiformis*, lane 4: S1wt treated by Endoglycosidase F1 from *Elizabethkingia miricola.* α–Mannosidase leaves three or more carbohydrate units at every N–glycosylation site. Endo F1 leaves only one carbohydrate unit (N–acetyl–D–glucosamine) for every N–glycosylation site. Successful deglycosylation can be seen by the difference in the resulting S1 nuclease mass. (**b**) IEF analysis. Lane loading is the same as in (a) except for the marker lane: IEF Marker 3–10. Determined values of the isoelectric point (~3.6) for S1wt and both deglycosylated versions are the same. (**c**) DLS analysis. Both deglycosylated versions behave similar to S1wt in the storage buffer and they are monomeric. The measured hydrodynamic radius is 2.49 ± 0.70 nm for S1wt, 2.47 ± 0.89 nm for S1–α–mann, and 2.23 ± 0.69 nm for S1–Endo F1. The apparent trend of decrease of hydrodynamic radius by deglycosylation cannot be reliably interpreted due to the observed experimental errors. (**d**) The activity of S1wt and S1 treated with Endoglycosidase F1 against ssDNA and RNA. Activity is reported as a change of absorbance at 260 nm over time normalized to the amount of enzyme used. S1wt and S1–Endo F1 display similar activity taking into account the decrease of enzyme mass by about 18% by deglycosylation and also the experimental error.

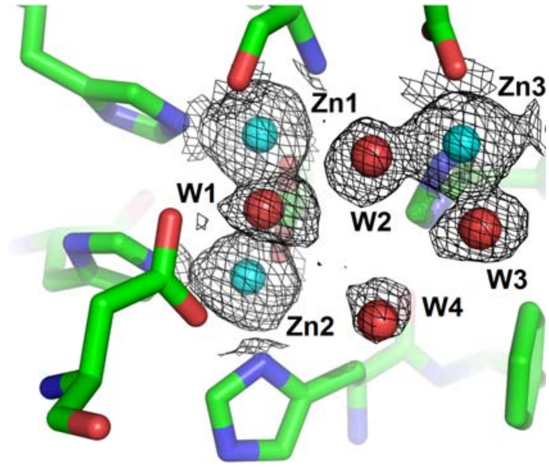

Figure B. The catalytic zinc cluster of the structure 5FB9 – unoccupied with bound water molecules. Zinc ions are shown as light blue spheres and water molecules as red spheres. The composite omit *2mF$_o$-DF$_c$* electron density map was calculated using *Phenix* [4] with the refinement mode. It is shown as a black mesh and contoured at a 1.5 σ level around the zinc cluster and water molecules present in the cluster. The four water molecules labelled W1–W4 are displaced and/or substituted upon binding of the various ligands in the other structures of this study. Their presence in the unoccupied active site is confirmed by the composite omit map. Molecular graphics were created using *PyMOL* (Schrödinger, LLC) and chain A of the structure 5FB9.

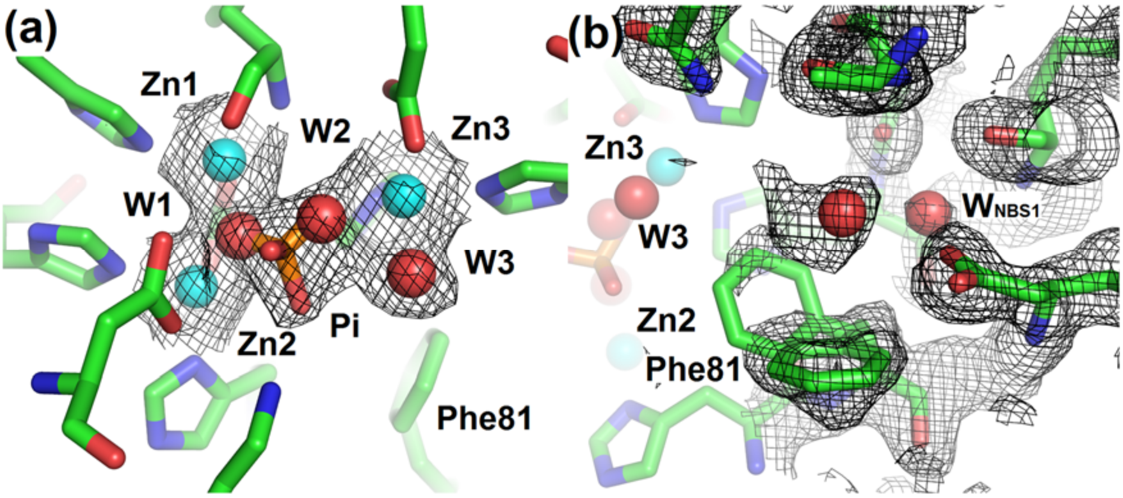

Figure C. The active site of the structure 5FBA – phosphate. Zinc ions are shown as light blue spheres, water molecules as red spheres, and phosphate as orange/red sticks. The composite omit *2mF$_o$-DF$_c$* electron density map was calculated using *Phenix* [4] with the refinement mode. It is shown as a black mesh and contoured at a 1.0 σ level around the zinc cluster, NBS1 and ligands of interest. (**a**) The presence of a phosphate ion (labelled Pi) in the catalytic zinc cluster is confirmed by the composite omit electron density. Based on behavior in the refinement, the phosphate ion was modelled with half occupancy as well as water molecules W1 and W2. (**b**) The nucleoside binding site (NBS1) with disordered Phe81. Molecular graphics were created using *PyMOL* (Schrödinger, LLC).

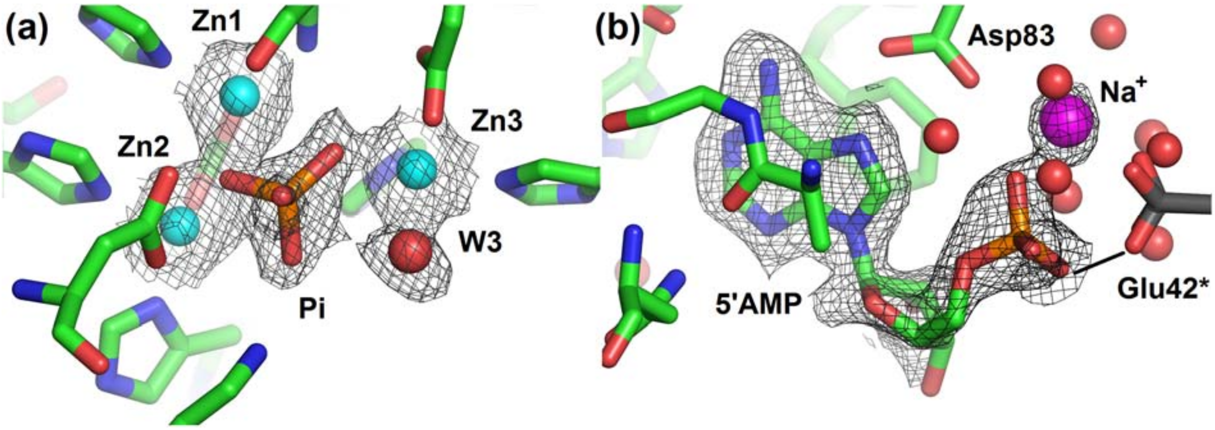

Figure D. The active site of the structure 5FBB – inhibitors with phosphate ion and adenosine 5'–monophosphate. Zinc ions are shown as light blue spheres and water molecules as red spheres. Phosphate is shown as orange/red sticks. The composite omit $2mF_o$-DF_c electron density map was calculated using *Phenix* [4] with the refinement mode. It is shown as a black mesh and contoured at a 1.0 σ level around the zinc cluster and ligands of interest. (**a**) The presence of the phosphate ion (labelled Pi) in the zinc cluster is confirmed by the composite omit electron density. (**b**) NBS1 with adenosine 5'–monophosphate. The presence of 5'AMP and a sodium ion is confirmed by the composite omit electron density. The presence of a sodium ion (magenta sphere) is supported by the coordination distances and behavior in the refinement. 5'AMP interacts with the sodium ion through the phosphate group and through this sodium ion also with Asp83 of NBS1. The sodium ion has no direct contact with symmetry–related protein molecules. The phosphate moiety of 5'AMP also interacts with Glu42 (shown in sticks, carbon – dark grey, marked by *) from a symmetry–related protein chain. This is the only direct contact of the ligand with a symmetry–related molecule. Molecular graphics were created using *PyMOL* (Schrödinger, LLC) and chain A of the structure 5FBB.

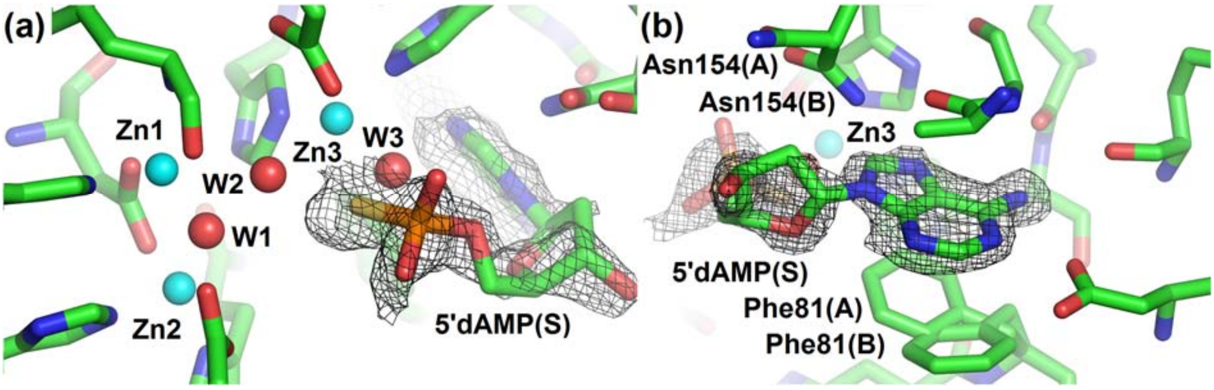

Figure E. The active site of the structure 5FBC – remodeled with 2'–deoxyadenosine 5'–thio–monophosphate. Zinc ions are shown as light blue spheres, water molecules as red spheres, and thiophosphate as yellow/orange/red sticks. The composite omit $2mF_o\text{-}DF_c$ electron density map was calculated using *Phenix* [4] with the refinement mode. It is shown as a black mesh and contoured at a 1.0 σ level only around the ligand of interest. 5'dAMP(S) in the structure is a product of cleavage of thiophosphorylated 2'–deoxyadenosine dinucleotide present in the crystallization experiment. (**a**) Interaction of the thiophosphate moiety of 5'dAMP(S) (sulfur – yellow, phosphorus – orange) with the zinc cluster. The orientation of the thiophosphate moiety with the sulfur atom interacting with Zn3 is confirmed by the intensity of the electron density peak at this position and also by the distance of this peak to the peak for phosphorus. 5'dAMP(S) was modelled with occupancy 0.8 due to the observed disorder in NBS1 (see panel b). Water molecule W3 was modelled with occupancy 0.2. (**b**) The nucleobase binding site (NBS1) with 5'dAMP(S). The presence of 5'dAMP(S) is confirmed by the composite omit map. The observed binding mode of 5’dAMP(S) is only compatible with Asn154 and Phe81 in alternative A, so it was modelled with occupancy 0.8 to interpret the observed disorder. The adenine moiety is slightly deformed after refinement of the structure, which is caused by the disorder in NBS1. The refinement procedure itself forces this deformation. Nevertheless, deformation of the base is insignificant for the interpretation of the binding mode. Molecular graphics were created using *PyMOL* (Schrödinger, LLC).

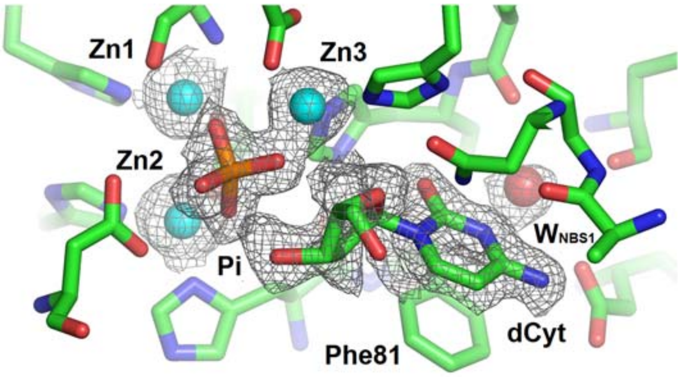

Figure F. The active site of the structure 5FBD – nucleotidase products with phosphate ion and 2'–deoxycytidine. The presence of the phosphate ion (orange/red sticks, labelled Pi) and 2'–deoxycytidine (labelled dCyt) is confirmed by the composite omit $2mF_o\text{-}DF_c$ electron density map (black mesh). 2'–deoxycytidine is a product of cleavage of thiophosphorylated 2'–deoxycytidine dinucleotide which was present in the crystallization experiment. Zinc ions are shown as light blue spheres, water molecules as red spheres, and phosphate as orange/red sticks. The composite omit $2mF_o\text{-}DF_c$ electron density map was calculated using *Phenix* [4] with the refinement mode. It is shown as a black mesh and contoured at a 1.0 σ level only around the ligands of interest and the zinc cluster. Molecular graphics were created using *PyMOL* (Schrödinger, LLC).

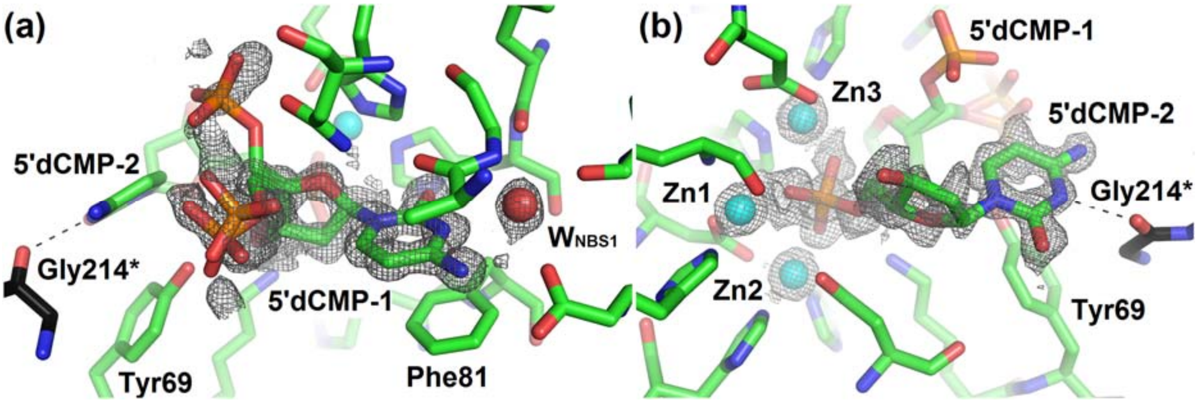

Figure G. The active site of the structure 5FBF – nuclease products with two molecules of 2'–deoxycytidine 5'–monophosphate. The catalytic zinc ions are shown as light blue spheres, water molecules as red spheres, and the phosphate moiety as orange/red sticks. The composite omit $2mF_o\text{-}DF_c$ electron density map was calculated using *Phenix* [4] with the refinement mode. It is shown as a black mesh and contoured at a 1.0 σ level only around the zinc cluster and the ligands of interest. The presence of both molecules of 2'–deoxycytidine 5'–monophosphate (labelled 5'dCMP) is confirmed by the composite omit $2mF_o\text{-}DF_c$ electron density map. (**a**) One molecule of 5'dCMP interacts with NBS1 (in the compact form) in the shallow binding mode, similar to 2'–deoxycytidine in the structure 5FBD – nucleotidase products (Figure F). The phosphate and 2'–deoxycytidine moieties of 5'dCMP in position -1 are modelled in three possible conformations (modelled based on $mF_o\text{-}DF_c$ and refined) with the main differences in the positions of the phosphate moiety. (**b**) The second molecule of 5'dCMP binds inside the zinc cluster via its phosphate group. The cytosine moiety only interacts directly with the symmetry–related protein chain (Gly214*, shown as sticks, carbon black) and so participates in the crystal contact. The cytosine moiety is also potentially involved in an interaction with Tyr69. The phosphate group binds inside the zinc cluster in the

same mode as the phosphate ion in the case of 5FBD – nucleotidase products (Figure F). Molecular graphics were created using *PyMOL* (Schrödinger, LLC).

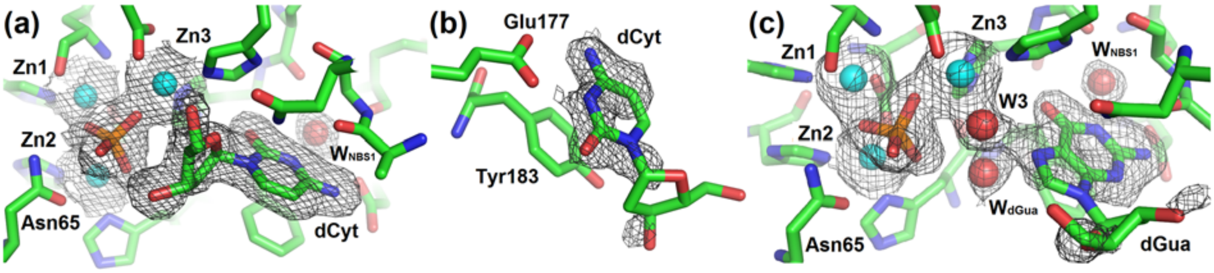

Figure H. The active sites and the Half–Tyr site of the structure 5FBG – mutant with products with the phosphate ion, 2'–deoxycytidine, and 2'–deoxyguanosine. Zinc ions are shown as light blue spheres, water molecules as red spheres, and phosphate ions as orange/red sticks. The composite omit $2mF_o\text{-}DF_c$ electron density map was calculated using *Phenix* [4] with the refinement mode. It is shown as a black mesh and contoured at a 1.0 σ level only around the zinc cluster and the ligands of interest. Only inorganic phosphate, 2'–deoxycytidine (dCyt), and 2'–deoxyguanosine (dGua) could be clearly identified in electron density and built. Both protein chains of the asymmetric unit have inorganic phosphate inside the zinc cluster but in slightly different orientations with respect to the zinc ions. The active site of chain A contains one molecule of dCyt whereas chain B one molecule of dGua. One additional molecule of dCyt binds in a site on the surface of chain A, near the active site. **(a)** The active site of chain A. Presence of the phosphate ion (orange/red sticks) and 2'–deoxycytidine (labelled dCyt) is confirmed by the composite omit $2mF_o\text{-}DF_c$ electron density map. **(b)** dCyt interacting with the Half–Tyr site. Electron density with a similar shape at this site can be observed also in chain B. It was not interpreted for its lower clarity. **(c)** The active site of chain B. Presence of the phosphate ion (orange/red sticks), 2'–deoxyguanosine (labelled dGua), and water molecules participating in the interaction of the guanine moiety with the zinc cluster (red spheres, labelled as W3 and W_{dGua}) is confirmed by the composite omit $2mF_o\text{-}DF_c$ electron density map. It is not clear whether the observed ligand is 2'–deoxyguanosine or 2'–deoxyguanosine 5'–monophosphate due to the disorder of the 2'–deoxyribose moiety. We chose to model this ligand as 2'–deoxyguanosine because the presence of the phosphate moiety is not supported by electron density. Molecular graphics were created using *PyMOL* (Schrödinger, LLC).

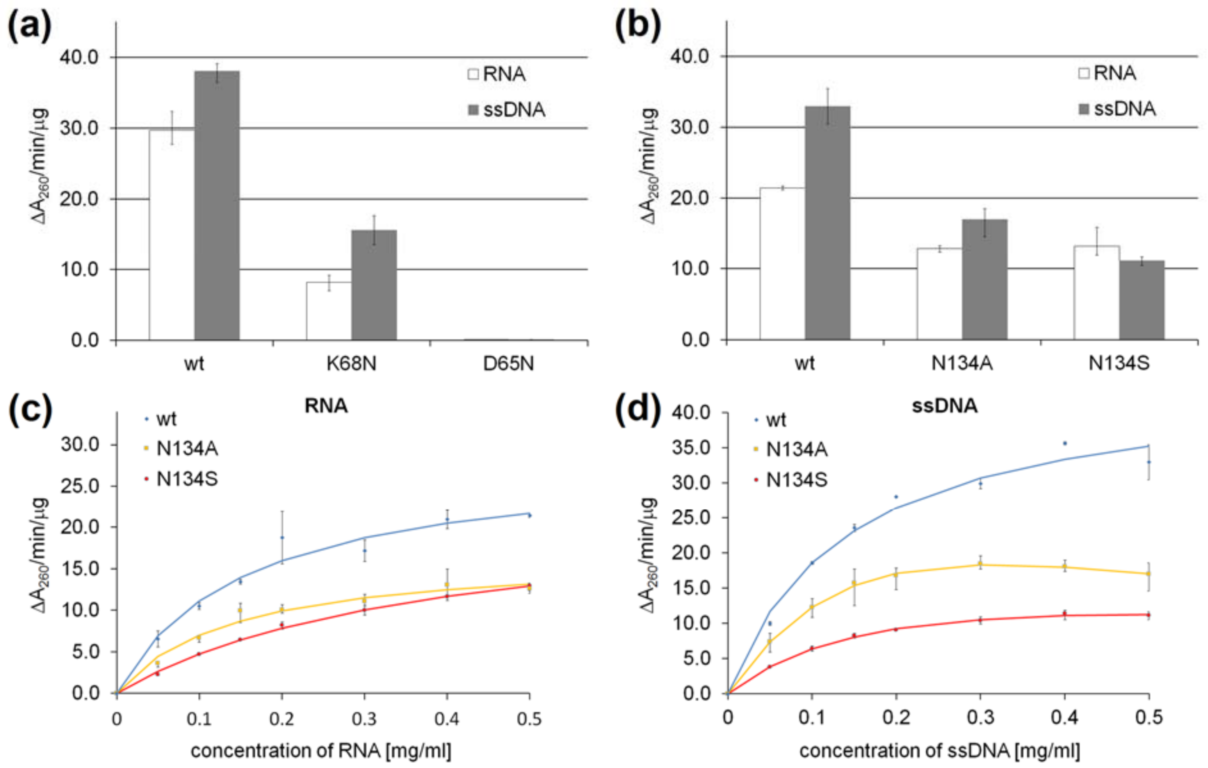

Figure I. Comparison of the catalytic activity of S1 wild type and mutants. (a) and **(b)** Activity was measured under standard reaction conditions and is reported as a change of absorbance at 260 nm over time normalized to the amount of enzyme used. Mutants reported in panels (a) and (b) were not measured at the same time. D65N and K68N were successfully expressed and purified earlier than N134A and N134S. **(c)** and **(d)** Comparison of kinetic parameters of nuclease S1 wild type and its mutants N154S and N154A using ssDNA and RNA as a substrate.

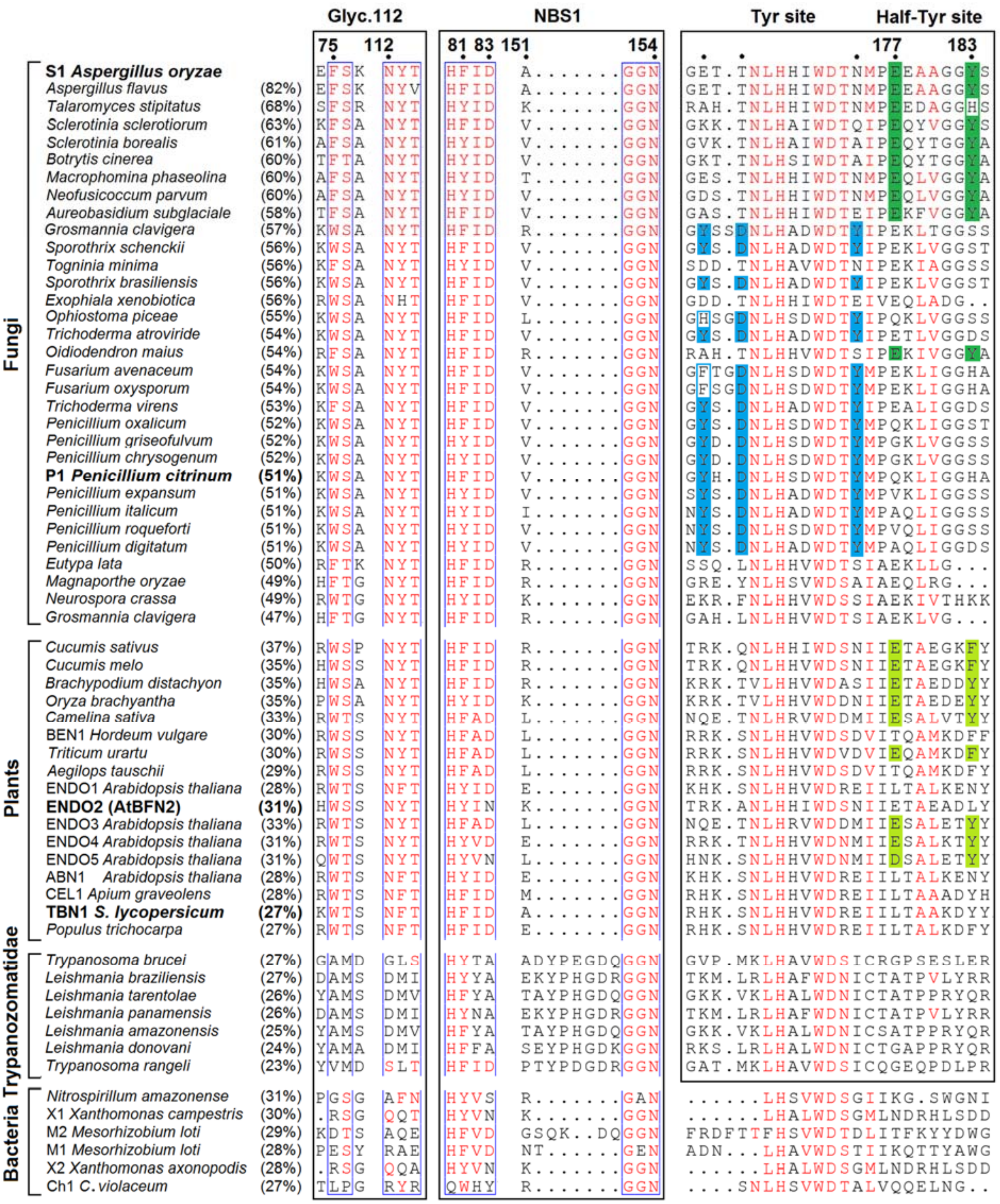

Figure J. Conservation of selected features in the S1–P1 nuclease family. Homologs of S1 nuclease were identified using an NCBI BLAST search [5]. Due to the high number of found homologs the list presented here was manually edited based on the intended demonstration of the selected features. Most of the bacterial homologs were selected based on Pimkin *et al.* [6]. The sequences were aligned using ClustalW2 [7]. Names of the enzymes with known structure are in bold characters. The column following the name shows sequence identity to S1 nuclease. The figure was created using ESPript [8] and edited. (**Glyc112**) Glycosylation site at position 112 along with its interacting partner, an aromatic amino acid at position 75. (**NBS1**) Alignment of selected residues involved in the formation of NBS1. The side chain of the residue at position 81 is involved in the stacking interaction with a nucleobase. The side

chain of the amino acid at position 83 provides hydrogen bonding to a nucleobase. Asp or Asn is conserved at position 83 in fungi and plants. In trypanozomatidae and bacteria this pattern is often broken. However, without the structure of such nuclease it is hard to estimate whether the function of the hydrogen bond partner is substituted by another amino acid, or nucleobase binding is facilitated in a different way. The second π–system donor is the peptide bond between the residue 151 and Gly152. Asn154 is always conserved. (**Tyr site and Half–Tyr site**) Residues involved in the formation of the P1 nuclease Tyr site are in the light blue box. Residues involved in the formation of the S1 nuclease Half–Tyr site are in the green box. These two sites are conserved only in fungi. Moreover, several S1–P1 like nucleases from fungi apparently do not possess either site. Residues with a possible role in the Half–Tyr site in plants are in the light green box. However, it is not possible to confirm the role of these residues in plants without the corresponding structures.

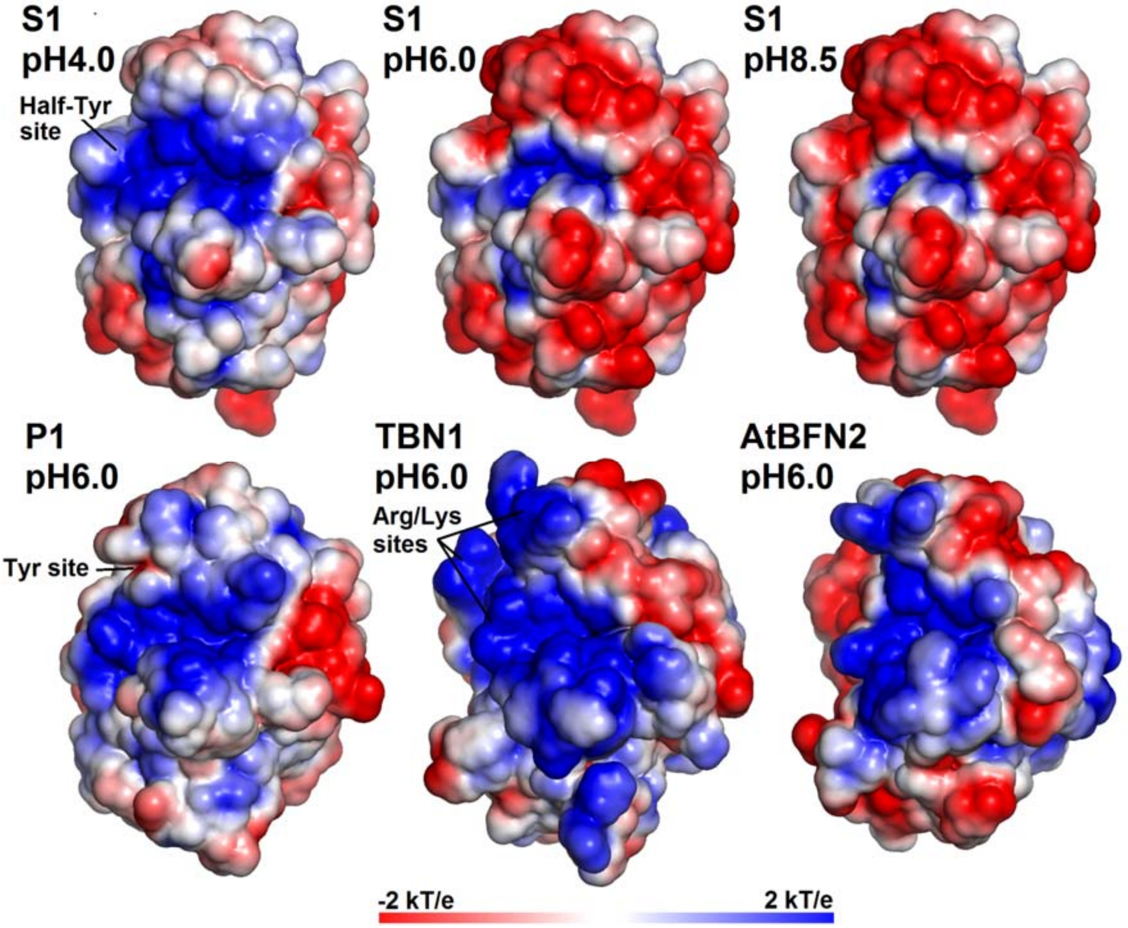

Figure K. Electrostatic potential distribution on the solvent accessible surface of the S1–P1 nuclease family members with known structure. Electrostatic potential distribution for S1 nuclease was calculated for protonation states at pH 4.0, pH 6.0, and pH 8.5 using the structure 5FBF – nuclease products. In the cases of P1 (PDB ID: 1AK0 [9]), TBN1 (PDB ID: 3SNG [10]), and AtBFN2 (PDB ID: 4CXO [11]) the electrostatic potential distribution was calculated only at pH 6.0 (close to their pH optimum of activity). All structures are shown in the same orientation with respect to the zinc cluster. Molecular graphics were created using *PyMOL* (Schrödinger, LLC).

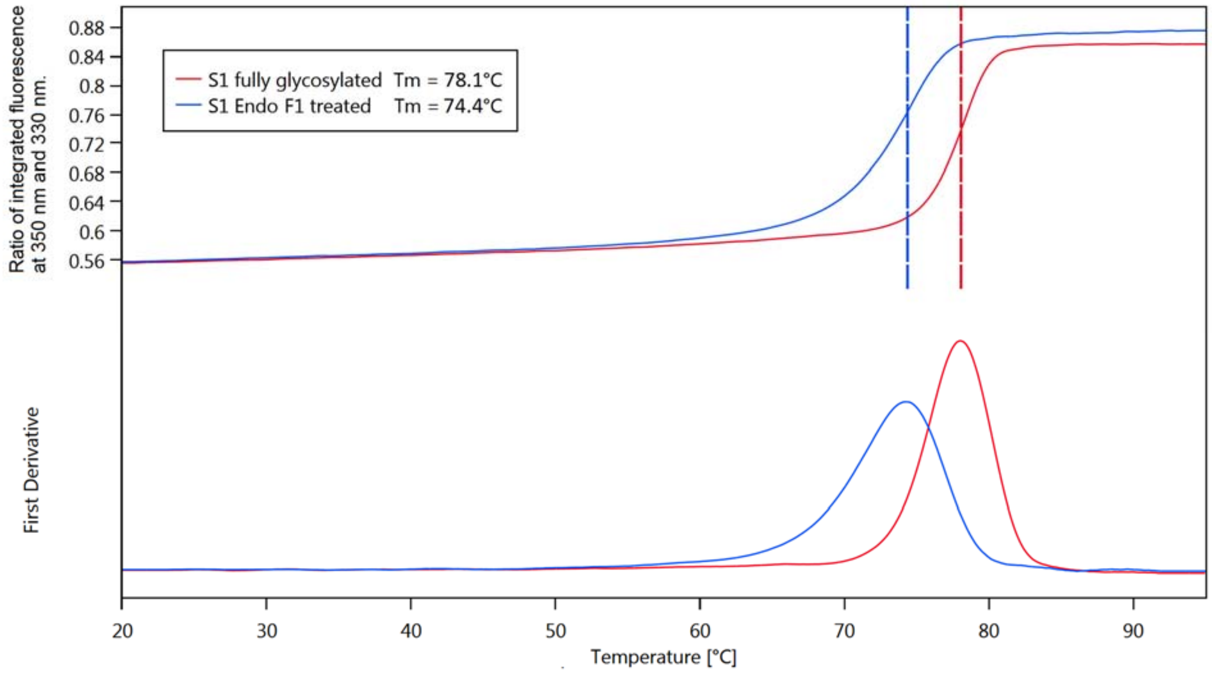

Figure L. Thermal stability of fully glycosylated S1 nuclease (red) and S1 nuclease treated with Endoglycosidase F1 (blue) measured by DSF. Measurements were performed in 25 mM Bis–Tris pH 6.0 with the addition of 50 mM NaCl, with protein concentration 0.5 mg/ml, and in temperature range 20 – 95 °C.

References

1. Baker NA, Sept D, Joseph S, Holst MJ, McCammon JA. Electrostatics of nanosystems: application to microtubules and the ribosome. Proc Natl Acad Sci U.S.A. 2001;98:10037–10041.
2. Dolinsky TJ, Czodrowski P, Li H, Nielsen JE, Jensen JH, Klebe G, Baker NA. PDB2PQR: Expanding and upgrading automated preparation of biomolecular structures for molecular simulations. Nucleic Acids Res. 2007;35:W522–525.
3. Søndergaard CR, Olsson MHM, Rostkowski M, Jensen JH. Improved treatment of ligands and coupling effects in empirical calculation and rationalization of pKa values. Chem Theor Comput. 2001;7:2289–2295.
4. Afonine PV, Grosse-Kunstleve RW, Echols N, Headd JJ, Moriarty NW, Mustyakimov M, et al. Towards automated crystallographic structure refinement with phenix.refine. Acta Crystallogr. 2012 D68:352–367.
5. Boratyn GM, Camacho C, Cooper PS, Coulouris G, Fong A, Ma N, et al. BLAST: a more efficient report with usability improvements. Nucleic Acids Res. 2013;41(W1):W29–W33.
6. Pimkin M, Miller CG, Blakesley L, Oleykowski CA, Kodali NS, Yeung AT. Characterization of a periplasmic S1–like nuclease coded by the *Mesorhizobium loti* symbiosis island. Biochem Biophys Res Commun. 2006;343:77–84.
7. McWilliam H, Li W, Uludag M, Squizzato S, Park YM, Buso N, et al. Analysis Tool Web Services from the EMBL-EBI. Nucleic Acids Res. 2013;41(W1):W597–W600.
8. Robert X, Gouet P. Deciphering key features in protein structures with the new ENDscript server. Nucleic Acids Res. 2014;42(W1):W320–W324.
9. Romier C, Dominguez R, Lahm A, Dahl O, Suck D. Recognition of single-stranded DNA by nuclease P1: high resolution crystal structures of complexes with substrate analogs. Proteins. 1998;32:414–424.
10. Koval T, Lipovova P, Podzimek T, Matousek J, Duskova J, Skalova T, et al. Plant multifunctional nuclease TBN1 with unexpected phospholipase activity: structural study and reaction-mechanism analysis. Acta Crystallogr. 2013;D69:213–226.
11. Yu TF, Maestre-Reyna M, Ko CY, Ko TP, Sun YJ, Lin TY, et al. Structural Insights of the ssDNA Binding Site in the Multifunctional Endonuclease AtBFN2 from *Arabidopsis thaliana*. PLoS ONE. 2014;9(8):e105821.
